# Studies of Genetic and Proteomic Risk Factors of Amyotrophic Lateral Sclerosis Inspire Biomarker Development and Gene Therapy

**DOI:** 10.3390/cells12151948

**Published:** 2023-07-27

**Authors:** Eva Bagyinszky, John Hulme, Seong Soo A. An

**Affiliations:** 1 Graduate School of Environment Department of Industrial and Environmental Engineering, Gachon University, Seongnam-si 13120, Republic of Korea; eva85@gachon.ac.kr; 2 Department of Bionano Technology, Gachon University, Seongnam-si 13120, Republic of Korea

**Keywords:** amyotrophic lateral sclerosis, biomarkers, genetic factors, proteomic markers, therapy, diagnosis

## Abstract

Amyotrophic lateral sclerosis (ALS) is an incurable neurodegenerative disease affecting the upper and lower motor neurons, leading to muscle weakness, motor impairments, disabilities and death. Approximately 5–10% of ALS cases are associated with positive family history (familial ALS or fALS), whilst the remainder are sporadic (sporadic ALS, sALS). At least 50 genes have been identified as causative or risk factors for ALS. Established pathogenic variants include superoxide dismutase type 1 (*SOD1*), chromosome 9 open reading frame 72 (*c9orf72*), TAR DNA Binding Protein (*TARDBP*), and Fused In Sarcoma (*FUS*); additional ALS-related genes including Charged Multivesicular Body Protein 2B (*CHMP2B*), Senataxin (*SETX*), Sequestosome 1 (*SQSTM1*), TANK Binding Kinase 1 (*TBK1*) and NIMA Related Kinase 1 (*NEK1*), have been identified. Mutations in these genes could impair different mechanisms, including vesicle transport, autophagy, and cytoskeletal or mitochondrial functions. So far, there is no effective therapy against ALS. Thus, early diagnosis and disease risk predictions remain one of the best options against ALS symptomologies. Proteomic biomarkers, microRNAs, and extracellular vehicles (EVs) serve as promising tools for disease diagnosis or progression assessment. These markers are relatively easy to obtain from blood or cerebrospinal fluids and can be used to identify potential genetic causative and risk factors even in the preclinical stage before symptoms appear. In addition, antisense oligonucleotides and RNA gene therapies have successfully been employed against other diseases, such as childhood-onset spinal muscular atrophy (SMA), which could also give hope to ALS patients. Therefore, an effective gene and biomarker panel should be generated for potentially “at risk” individuals to provide timely interventions and better treatment outcomes for ALS patients as soon as possible.

## 1. Introduction

Amyotrophic lateral sclerosis (ALS), or “Lou Gehrig’s disease”, is a neurodegenerative disorder resulting in the degeneration of motor neurons inside the brain and spinal cord. The disease affects lower motor neurons (LMNs) in the brainstem and spinal cord and upper motor neurons (UMNs) in the motor cortex [1,2]. ALS patients also present additional symptoms, including cognitive decline and personality changes [3,4]. Disease phenotypes usually appear in mid- or later life (50s–70s) but occasionally occur in earlier adulthood (under 30) and to a lesser degree in elderly patients (over 80) [5]. The majority of patients die 3–5 years after the first disease symptoms, whilst a juvenile minority can exhibit prolonged survival (more than 10 years) [6]. Most ALS cases are sporadic, but approximately 5–10% of disease cases are associated with a positive family history of the disease. Disease inheritance can be autosomal dominant, recessive, de novo and X-linked (XL). ALS is a genetically complex disease with >50 genes associated with disease onset. The disease usually starts with muscle issues such as tightness, weakness, and cramps, resulting in patients exhibiting reduced grip strength and poor balance. Initial symptoms can manifest in one of several body parts, such as bulbar-onset, arm-onset, or leg-onset. As the disease progresses, the facial muscles lose coordination, making speaking, breathing, and swallowing increasingly difficult. In the later stages, paralysis usually ensues, with patients losing the ability to walk unaided, rendering them entirely bedridden. Since the disease also affects the respiratory muscles, respiratory failure is often the leading cause of death [3,7]. In this regard, ALS and frontotemporal dementia (FTD) share several clinical, genetic, and pathological features. Approximately half of ALS patients develop FTD-like phenotypes (cognitive decline, personality changes), and 30% of FTD patients go on to develop motor neuron impairments [8,9,10]. 

**Figure 1:**
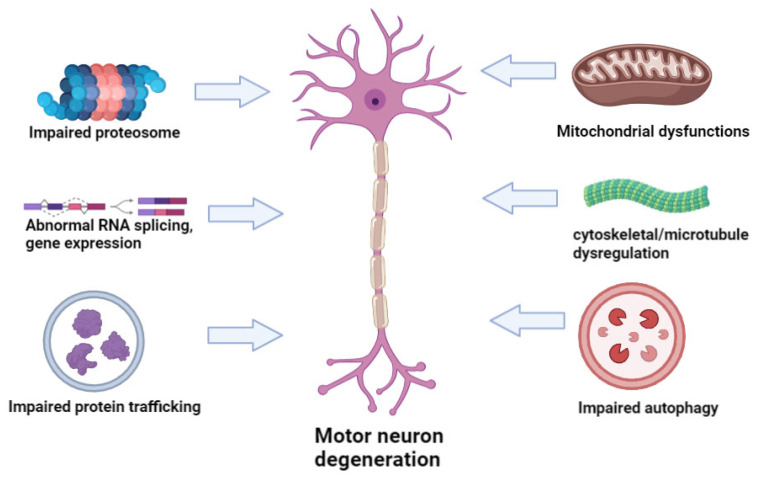
Pathological processes that may contribute to neuronal death in MND and ALS.

Several genes have been identified which could impact both FTD and ALS, including chromosome 9 open reading frame 72 (*c9orf72*), TAR DNA Binding Protein (*TARDBP*), Sequestosome 1 (*SQSTM1*), Valosin Containing Protein (*VCP*), Fused In Sarcoma (*FUS*), TANK Binding Kinase 1 (*TBK1*), or Cyclin F (*CCNF*) [9]. However, the genetic overlap among sporadic forms of FTD and ALS remains poorly understood. A recent clinical study by Karch et al. further highlighted this problem with the discovery of ALS loci rs13302855 and rs3849942 (nearest gene, *C9orf72*; *p* = 0.03 for rs13302855 and *p* = 0.005 for rs3849942) [11] with the authors concluding sALS is a selectively pleiotropic, polygenic disorder. Besides FTD, ALS-related genes are considered risk factors for other neurodegenerative diseases, such as Charcot–Marie–Tooth (CMT), Parkinson’s disease (PD), Alzheimer’s disease (AD), bipolar disease (BD), motor neuron diseases (MNDs), Paget’s disease, Inclusion-Body Myositis (IBM), Huntington’s disease (HD), Hereditary spastic paraplegia or ataxia [10,12]. Furthermore, additional genes can also exacerbate non-neurodegenerative disease phenotypes, such as glaucoma (optineurin, *OPTN*) [13], Enterovirus-induced Type-1 diabetes (Peripherin, *PRPH*) [14], leukemia, or cancer (TATA-box-binding protein-associated factor 15, *TAF15*) [15,16]. The above mutations are thought to impair multiple pathways, such as proteasome functions, RNA splicing, protein trafficking, autophagy, microtubule organization or mitochondrial pathways, leading to motor neuron degeneration and ALS progression (Figure 1). 

## 2. Genes and Molecular Mechanisms Contributing to ALS

### Primary Causative Genes and Function

To date, there are four mendelian genes that cause ALS: The Cu–Zn superoxide dismutase (*SOD1*), TAR DNA Binding Protein (*TARDBP*), Fused in Sarcoma (*FUS*) gene and Chromosome 9 Open Reading Frame 72 (*c9orf72*) (Table 1). Since its identification 30 years ago, almost 200 *SOD1* mutations have been attributed to familial and sporadic forms of ALS; most of the disease-causing variants are missense mutations and comprise 15–30% of inherited ALS cases and less than 2% of sporadic ALS cases [17]. The *TARDBP* gene constitutes 4.2% and 1.5% of European and Asian fALS cases, respectively, whereas *FUS* is more common in Asian than European fALS cases (6.4% and 2.8%, respectively). Finally, the *c9orf72* gene is common in European familial ALS cases (more than 30%), while it may be a rare causative factor among Asian fALS cases (less than 3%). However, these genes are rarely encountered in either European or Asian (less than 8% and 3%, respectively) sporadic ALS (sALS) cases [18].

The *SOD1* gene is located on chromosome 21, producing a 153 conserved-amino-acid-long protein in the cytoplasm, nucleus and mitochondria. In the active configuration, the protein exists as a homodimer enzyme containing eight antiparallel beta strands housing copper and zinc ions, which play a role in the dimerization and disulfide bond stabilization (between Cys57 and Cys146). SOD1 protein functions as an antioxidant enzyme, which protects the cells from oxidative stress by the conversion of catalyzing toxic superoxide anions into hydrogen peroxide and oxygen molecules, lowering the steady state of radical ions (nitric oxide, superoxide, and peroxynitrite), regulating apoptosis [10,19,20,21]. SOD1 dysfunctions may also impact another motor disease, spastic tetraplegia and axial hypotonia (STAHP) [22]. Most *SOD1* mutations are heterozygous with autosomal dominant inheritance, but homozygous variants with autosomal recessive inheritance have been reported in rare cases [23]. *SOD1* mutations increase protein instability and the capacity to misfold due to metal ion and disulfide bond deficiencies. Collectively, such deficits can result in oligomerization, abnormal protein aggregation, and the formation of amyloid-like aggregates [24,25,26]. In addition, the potential prion-like properties of pathogenic *SOD1* can lead to nuclear and mitochondrial DNA damage [19], with resultant amyloid-like fibrils shown to be intracellularly transmissible [24]. *SOD1* variants disrupt cytosolic and mitochondrial calcium homeostasis, leading to abnormal cell functions and alterations of glial cell-motor and skeletal muscle-motor neuronal crosstalk, triggering apoptosis [27].

**Figure 2:**
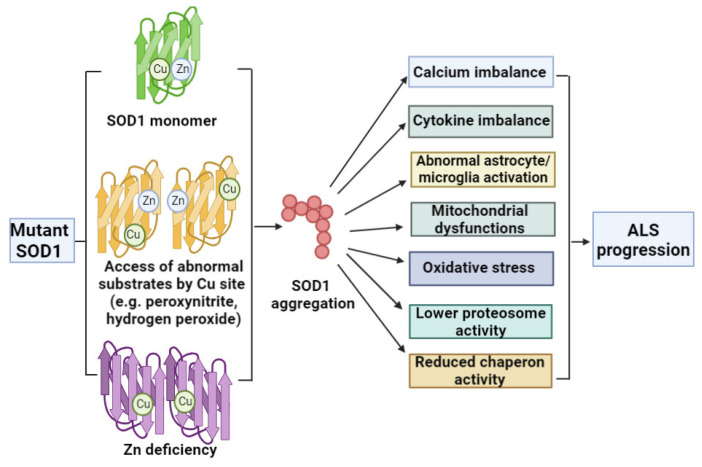
Mutant *SOD1* and ALS-related pathways, adapted from Refs. [20,21,27].

Several geographical mutations are associated with *SOD1*; for example, A4V is a common mutation in American populations, which is thought to accelerate progressive familial ALS, reducing disease duration to 2 years or less. The disease initially presents with limb and bulbar muscle weakness, primarily affecting the lower motor neurons [28,29]. Common among Japanese patients, H46R is a variant ALS-causing mutation associated with slower disease progression (up to 15 years). The H46R mutation is thought to impair the copper-binding site of *SOD1* [30,31,32]. Another common ALS mutation associated with rapid disease progression is *SOD1* G93A. Transgenic mice carrying this variant are frequently used in ALS drug screening [33,34]. Figure 2 summarizes the possible impact of *SOD1* mutations (including A4V or H46R) in ALS.

**Figure 3:**
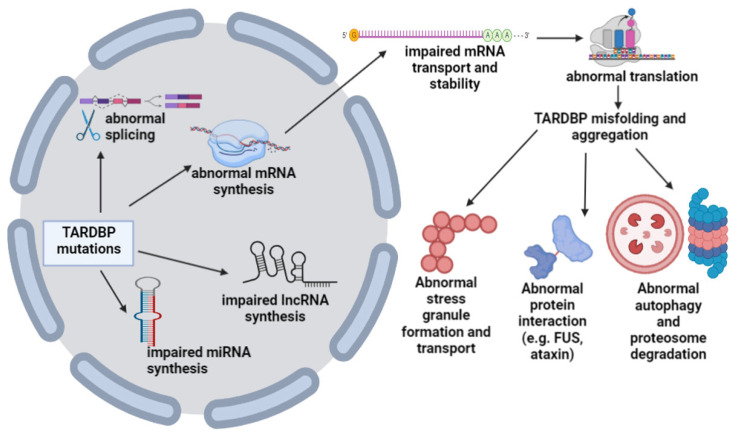
TARDBP mechanisms in ALS progression, adapted from Refs. [46,47,48].

In 2006, prion-like mutations were identified in the C-terminus-encoding regions of the *TARDBP* gene and deemed causal for autosomal dominant ALS and associated TDP43 proteinopathy. The *TARDBP* gene contains six exons on chromosome 1, expressed ubiquitously, residing mainly in the nucleus [35]. The TDP43 protein encoded by this gene is essential for RNA transcriptional regulation, splicing and stabilization, potentially impacting metabolism [36,37]. In addition, TARDBP protein could bind to different proteins (such as hnRNP proteins) and influence other protein–protein interactions [38], some of which are integral to mitochondrial function. Its overexpression and mislocation can lead to TDP43 proteinopathy, impairing mitochondrial functions [39]. The protein is 414 amino acids long, possessing several domains, including the N-terminal domain (between aa1 and 76), domain for nuclear localization signal (NLS; aa82–98), RNA recognition motif-1 (RRM1, aa106–176) and RNA recognition motif-2 (RRM2, aa191–259), and the C-terminal domain (aa 274–414). The RNA recognition motif-2 contains the specific sequence of nuclear export signal (aa239–250). The N-terminal domain is involved in the self-regulation of *TARDBP* transcription and RNA splicing through homodimer formation [40]. The RNA recognition motifs participate in numerous processes, such as the direct binding of DNA or RNA, splicing and translation, limiting oligomer formation and potential aggregation [40,41]. Finally, the C-terminal domain contains a prion-like domain between residues 274 and 414. This domain comprises polar amino acids essential in TDP43 aggregation [41,42,43]. The majority of ALS-related *TARDBP* mutations are associated with the misfolding aggregation of TDP43 (for example, Q331K, M337V, Q343R, N345K, R361S, G376D, or N390D) protein [44] through different mechanisms. For example, G376D is thought to increase the mislocalization of protein into the cytoplasm [45]. Furthermore, additional mutations, such as D169G or R361S, are associated with larger stress granular production and disease acceleration. Mutant *TARDBP* may result in impaired protein–protein interactions between TARDBP protein and its binding partners, such as FUS or ataxin. The TDP-43 aggregations may disturb the autophagic mechanisms and proteosome formation. Furthermore, mutations in *TARDBP* could impact the processing of miRNAs and lncRNAs (Figure 3) [46,47,48].

**Figure 4:**
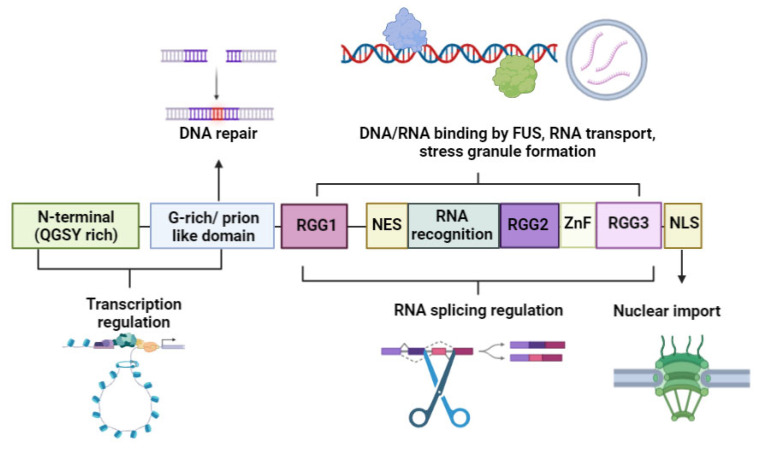
Domains of *FUS* gene and possible mechanisms of FUS mutations, adapted from Refs. [50,51].

The *FUS* gene could also be a significant causative gene for ALS. The initially suspected oncogene is located on chromosome 16 and encodes a nuclear RNA-binding protein. The respective protein has been implicated in RNA metabolism, transcription regulation, RNA transport, micro-RNA processing, and genome maintenance [49] and is considered a key player in neuronal homeostasis (neuronal plasticity and dendrite integrity) [50]. The protein is 526 amino acids long, containing several domains, including an N-terminal QGSY-rich region (1–165aa), two glycine-rich or prion-like domains (aa165–222 and aa391–407), with a nuclear export signal motif (NES, 280–377aa), three arginine-glycine rich (RGG) domain boxes (RGG1-aa222–280, RGG2-aa377–418, RGG3-aa454–501), zinc finger (aa418–454) and a C-terminal area, called proline-tyrosine nuclear localization signal (aa501–526). Inside RGG2, another prion-like domain was identified, which is located between residues 391 and 407 (Figure 4). Along with The RNA recognition domains zinc finger (Znf), motifs could be involved in FUS interaction with RNA. In addition, RGG domains are also implicated in nucleotide binding and other cellular pathways, such as cell division or the formation of stress granules. The N-terminal region and the glycine-rich (GC) domain may impact transcription regulation. Furthermore, the GC domain may also influence DNA repair mechanisms, nuclear localization signals, and importation [48,49,51,52].

A significant ratio of *FUS* mutant ALS cases is associated with an early-onset disease (under 40 years of age) [51,52], many of which are either gain-of-function or loss-of-function variants, influencing multiple pathways. FTD and ALS with *FUS* mutations may present TDP43 inclusions in their brain, but TDP43 proteinopathy can also be absent in ALS patients with *FUS*, particularly when the abnormal transcript utilizes TDP43-independent pathways [53]. For example, the postmortem brains of ALS patients can exhibit active p38 MAPK elevations, suggesting that aberrant expression may perturb the p38 MAPK pathway, leading to stress-induced neuronal loss [54]. Moreover, other *FUS* mutations are thought to exercise their toxicity by reducing ion channel expression and synaptic activity, inhibiting intra-axonal translation [55]. Furthermore, additional mutations such as R521G, R522G, or ΔExon15 may result in the abnormal splicing of *FUS* transcript and autoregulation of *FUS* levels [56]. As a result, the abnormal protein could induce nonsense-mediated decay (NMD) and promote the up-regulation of several pro-NMD factors (Up-Frameshift Suppressor 3 Homolog B, UPF3b; and UPF1 RNA Helicase and ATPase, UPF1) and the down-regulation of NMD inhibitors, such as UPF3a, leading to NMD hyperactivity and neurotoxicity [57]. Additional investigations have identified *FUS* immunoreactive cytoplasmatic inclusions inside neurons and glial cells. The inclusions usually occur in the nucleus [58], but can induce conformational changes in cytoplasmic proteins due to their prion-like domain, leading to further aggregation [59]. Other mutations include R521L, R521G, R521H, R521C, P525L, and R495Qfs527X or R495X, primarily associated with FUS protein mislocalization and mitochondrial impairment. Impairment can manifest as mitochondrial shortening and fragmentation, whilst the mutant R495X is thought to reduce the organelle’s size [60]. In addition, other *FUS* mutations may down-regulate genes (such as cyclooxygenases, COX) essential to oxidative phosphorylation, impacting skeletal muscle functions [61].

**Figure 5:**
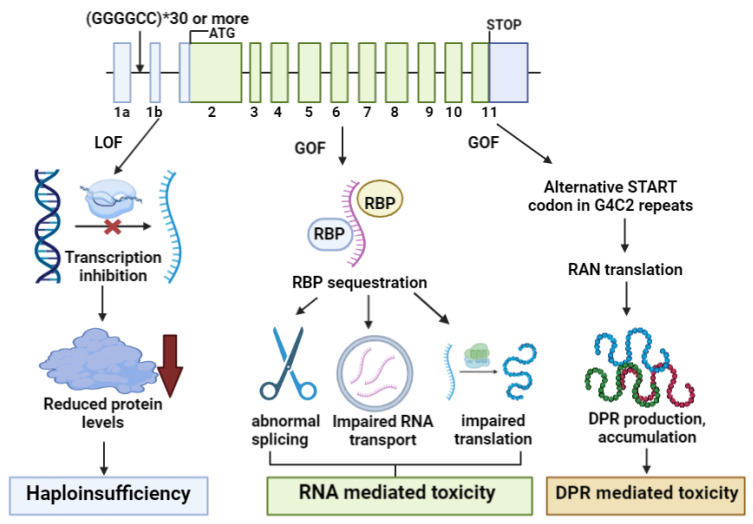
Activation of neurodegenerative pathways resulting from *C9orf72* repeat expansion. Repeat expansion could be associated with loss-of function (LOF) mechanism, called haploinsufficiency. Gain-of function (GOF)mechanisms may also be related with G4C2 expansion, such as RNA-Binding Proteins (RBP) sequestration (leading to alternative splicing and impaired RNA transport/translation). Additional GOF pathway could be an alternative START codon generation and Repeat Associated Non-AUG (RAN) translation, leading to dipeptide repeat (DPR) production, accumulation and toxicity. The figure was adapted from Refs. [66,67,68,69,70,71,72,73].

The next causative pathogenic variant is the hexanucleotide repeat expansion mutation (G4C2; (g.26724GGGGCC (3_23)) located between the non-coding exons 1a and 1b of the *c9orf72* gene. DeJesus Hernandez et al. (2011) revealed that c9orf72 was present in 11.7% of familial FTD and 23.5% of familial ALS patients. In healthy individuals, repeat expansions typically range from 3 to 30. G4C2 is the abbreviation of hexanucleotide repeat expansion mutation (HREMs) and ranges from 30 to 100 in ALS patients, although G4C2 expansions in the hundreds or thousands have been reported in sALS patients [62]. In a normal brain, the standard splicing of the c9orf72 transcript results in three different isoforms: (1) variant 1, which contains exon 1b and coding exons 2–11, but not exon 1a or the G4C2 repeats; (2) variant 2, which contains the exon 1a and repeat expansion, but only contains coding exons 2–5; and (3) variant 3, containing exon 1a, the repeat expansion region, and all coding exons. Variants 1 and 3 can manifest as a 481 amino-acid-long protein, while variant 2 was predicted to encode a 222 amino-acid-long protein. However, in the brains of FTD or ALS patients with repeat expansions, the *c9orf72* variant 1 is often undetectable. An initial study suggested that the repeat expansion could limit the expression *of c9orf72* variant 1, significantly impacting brain development and function [63,64,65]. Moreover, longer G4C2 expansions were suggested to alter the methylation pattern of the *c9orf72* gene and its promoter activity. Repeat expansions were associated with a more significant degree of methylation, leading to reduced transcriptional activity and haploinsufficiency, resulting in dipeptide repeat protein (DPR) and glutamate receptor elevations. Furthermore, intermediate repeats, ranging between 7 and 24 G4C2 units, showed a slight increase in methylation compared to a low number of repeats (2–6), suggesting that “intermediate alleles “may also act as risk alleles [65,66]. Even though *c9orf72* participates in loss-of-function (LOF) mechanisms and has the potential for gain-of-function toxicity (GOF) (Figure 5), the variants alone are not considered a primary cause of motor degeneration [67]. Another factor potentially contributing to *c9orf72*-related disorders is the translation of expanded intronic G4C2 through an alternative START codon (such as Kozak-sequence- or repeat-associated non-ATG-initiated translation). The abnormal translation of G4C2 repeats could result in DPR, such as poly-(Gly-Ala or polyGA), poly-(Gly-Pro or polyGP) and poly-(Gly-Arg or polyGR) proteins, which have a strong tendency to aggregate due to their high degree of hydrophobicity. DPR aggregates are moderately neurotoxic, whilst poly-GA aggregates are highly neurotoxic [63,64,65,68,69,70,71]. However, the exact mechanism by which said aggregates contribute to neuronal loss in FTD/ALS patients remains to be elucidated. Another consequence of c9orf72-related expansion is the generation of sense and antisense RNA foci. RNA foci exist in several brain regions (cerebellum, motor and frontal cortex, or hippocampus), sequestering binding proteins and associated RNA processing machinery [71]. Expansion may also increase small ubiquitin/p62 positive (but TDP43 negative) aggregates in different neuroanatomical regions, such as the hippocampus, cerebral cortex, or neocortex, limiting p62 controlled autophagy (arginine methylation initiation) and the removable of stress granules [72]. Furthermore, *c9orf72* repeat expansion could impair the function of master DNA repair kinases, contributing to ataxia telangiectasia (AT). In addition, perturbation of kinase functions due to *C9orf72* repeat expansion might lead to heterochromatin formation and an increase in the frequency of double-strand DNA breaks [73]. 

## 3. Additional Rare ALS-Related Genes

**Figure 6:**
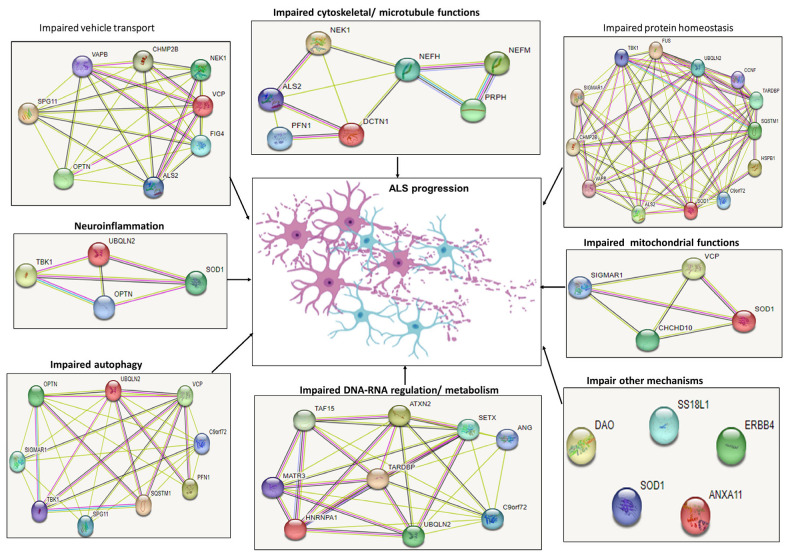
Potential biological processes/functions affected by common and rare ALS-related genes during ALS progression [18,74]. The differently colored edges may represent the proterin-protein associations. The light blue means, association was verified from curated databases. Pink means, interactions were proven experimentally. The darker green means “gene neighbourhood”. Red means possible gene fusion. Darker blue means gene co-occurrence. Light green means “text mining” or possible association from literature. The black means that the genes may interact through co-expression. The purple edge means that these genes may share homology. Network analyses were performed by STRING software version 11.5 (https://string-db.org/, accessed on 20 July 2023).

Even though *SOD1*, *TARDBP*, *c9orf72*, and *FUS* mutations are relatively common in familial ALS, several additional genes were identified which could play a role in ALS onset. In addition, approximately 44.5% of European and ~60% of Asian fALS cases are associated with rare genetic factors. More than 50 genes (Table 2, Figure 6) have been associated with ALS, and this number is set to increase [18].

### 3.1. Genes Involved in Protein Trafficking

Charged multivesicular body protein 2 (*CHMP2B*) is on chromosome 3 and encodes the 213 amino-acid-long protein. This essential protein plays multiple roles in the Endosomal Sorting Complex Required For Transport III (ESCRT-III), from the biogenesis and sorting of endosomes to the trafficking of multivesicular bodies (MVBs), proteostasis, and neuronal homeostasis. *CHMP2B* mutations are not unique to ALS and have been associated with numerous diseases, including FTD and progressive muscular atrophy (PMA) [75,76]. However, the mutation in *CHMP2B* intron5 was recently implicated in the hyperphosphorylation of TDP-43 via the ubiquitin-proteasome system (UPS), decreasing ubiquitination and turnover of casein kinase 1 (CK1), highlighting a genetic link between *TARDBP* and *CHMP2B* and the associated ALS pathology [77]. 

Another gene implicated in ALS is the Valosin-containing protein (*VCP*) which exhibits chaperon-like properties that regulate the ubiquitin-proteasome system (UPS) and autophagic processes. *VCP* mutations are rare causative factors for ALS [78] and are associated with other diseases, such as inclusion body myopathy (IBM), Paget’s disease, and FTD. *VCP* plays a crucial role in UPS-mediated protein degradation processes, and its impairment leads to the accumulation of ubiquitinated proteins. However, the specific role of *VCP* in autophagy remains unclear, as the protein has been implicated in several processes, including autophagy activation, autophagic complex maturation, autophagosome, and lysosome fusion and homeostasis [79]. In addition to *VCP*, the vesicle-associated membrane-protein-associated protein B (*VAPB*) gene plays a role [80,81] in vesicle trafficking and calcium homeostasis, modulating neuromuscular junction activity. The observable impact of *VAPB* mutants (P56S) includes disorganized endoplasmic reticulum (ER) structures and an enhanced propensity of a cell to form aggregates [82,83]. Another rare gene implicated with ALS is phosphoinositide 5-phosphatase (*FIG4*), which regulates endosomal/lysosomal vesicle transport [84]. *FIG4* has a potential role in Charcot–Marie–Tooth disease, ALS, primary lateral sclerosis (PLS), recessively inherited hereditary motor and sensory neuropathy, or Yunis–Varón syndrome [84]. Furthermore, additional FIG4 mutations in ALS include I41T, F254Sfs*8 or Y647C, which can exhibit autosomal dominant inheritance patterns [85,86,87,88]. Spastacin (*SPG11*) is a primary causative factor for autosomal recessive hereditary spastic paraplegia (HSP). Still, it may also play a role in other neurodegenerative diseases, including autosomal recessive juvenile ALS and CMT. *SPG11* could play a role in multiple processes, including lysosome trafficking, cholesterol transport, autophagy, and calcium homeostasis. ALS’s *SPG11* mutations (such as c.704_705delAT and c.5199delA) may be associated with a lack of thinned corpus callosum and cognitive and ocular abnormalities [89,90]. NIPA Magnesium Transporter 1 (*NIPA1*) was initially associated with Prader–Willi/Angelman syndrome 1 or Type 6 HSP, but it may also impact ALS [91,92,93]. *NIPA1* impacts magnesium metabolism inside the cells. It may also affect endosome regulation inside neurons and epithelial cells [92]. The GCG repeat (or polyalanine repeat) expansion in the 5′ end of *NIPA1* was suggested to impact ALS risk. ALS patients with *NIPA1* CGG repeat expansions may be associated with increased ALS risk, earlier disease onset, and reduced survival [91,93]. The impact of *NIPA1* in ALS was verified by a large-scale replication analysis performed by Tazelaar et al. in 2019. The pathogenic mechanism of CGG repeat expansion remained unclear. The polyalanine repeats may result in abnormal protein conformation, protein–protein interactions, and aggregation [94].

### 3.2. Genes Influencing Proteosome/Chaperone Functions

Cyclin F (*CCNF*) gene encodes the cyclin F, which functions as ubiquitin-protein ligase. Cyclin F could regulate proteosome degradation by recruiting substrates fort polyubiquitination. *CCNF* mutations (such as S621G, S195R, K97R, S509P, I772T or S3G) could limit proteosome turnover and the post-translational modification of CCNF *protein* and E3 ligase activity [95,96,97]. For example, the S621G mutation might cause the CCNF phosphorylation site to adopt a less-than-optimal configuration, whereas S509P, K97R, S195R, or S621G might reduce or enhance E3 ubiquitin ligase activity [95,96,97,98].

Ubiquilin-2 (*UBQLN2*) is a member of the ubiquitin proteosome system and plays a role in the homeostasis of different proteins and the quality control of mitochondrial proteins. Also, *UBQLN2* participates in the degradation of ubiquitinated proteins through proteosome and autophagic pathways. Different mutations have been linked to FTD and ALS [99,100]. Specifically, ALS-related *UBQLN*2 mutations (such as R497H, P506S, and P509S) could restrict proteosome-mediated degradation due to their reduced binding ability, leading to aggregation and neurotoxicity [95]. Furthermore, mutations in *UBQLN2* may decrease the expression of ATPase H+ Transporting V1 Subunit G1 (ATP6v1g1), leading to impaired autophagy due to failed acidification and maturation [100,101]. 

The chaperon small heat-shock protein family B member 1 (*HSPB1*) mutations are thought to impact CMT and distal hereditary motor neuropathy and, therefore, considered a potential risk factor for ALS. *HSPB1* mutations are suspected to enhance protein misfolding and aggregation. For example, *HSPB1* mutations reported in sporadic ALS cases, such as Q190H and A204Gfs* 6, were assumed to reduce the protein stability and chaperon activity, rendering motor neurons more sensitive to stress. *HSPB1* mutations may also be associated with impaired autophagy by reducing the ability of Sequestosome 1 (SQSTM1)/p62 body formation [102,103,104,105].

### 3.3. Variants with the Potential to Impact Autophagic Pathways

Sequestosome 1 (*SQSTM1*) gene encodes the sequestosome 1/p62 protein, with suspected roles in FTD, Paget bone disease, or familial and sporadic forms of ALS. The *SQSTM1* mutations could impact the turnover of p62, promoting aggregation or autophagy, and impair its interaction with the Kelch-like ECH-associated protein 1 (KEAP1) [106]. For example, ALS-associated mutation L341V in SQSTM1, may impair autophagy by reducing SQSTM1 affinity for the lipid-anchored form of LC3 protein, impairing phagophore development and reducing cell survival [107]. Another mutation, G427R, may abolish an important phosphorylation site in serine 351, limiting interactions between KEAP1 and SQSTM1, reducing the expression of erythroid-derived 2 and Nuclear Factor Erythroid 2-Related Factor 2 (NFE2L2/Nrf2)-targeted genes, elevating the TDP-43 associated stress granule formation under oxidative stress [108]. 

TANK-binding kinase 1 (*TBK1*) is an NF-κB activator kinase protein that could impact ALS and FTD. TBK1 protein consists of a kinase, a ubiquitin-like, and two scaffold dimerization domains, permitting its participation in several pathways, including mitophagy (mitochondrial clearance) and autophagy by phosphorylating several adaptor proteins, such as optineurin (OPTN) or Parkin (PRKN). In addition, TBK1 may be involved in T-cell activation and autoimmunity [109,110]. *TBK1* mutations could potentially generate a variety of disease phenotypes via several mechanisms. For example, R47H, D135N, G217R, or R228H expression could result in lower autophosphorylation. Specifically, G217R could decrease mitochondrial fragmentation and the interaction between TBK1 and PRKN. Other mutations, such as G217R, R357Q, and M559R, could disrupt TBK1 assembly and mitophagy events. In addition, D135N or D559N mutations could impair mitophagy by inhibiting LC3 recruitment, increasing the likelihood of accumulation of damaged mitochondria. Moreover, these mutations could also elevate mitochondrial reactive oxygen species (ROS), promoting aggregate formations and neuronal cell death and neuroinflammation [110,111].

Optineurin (*OPTN*) was initially reported as a causative gene for glaucoma and was later found to contribute to ALS pathology. The gene is expressed in several human organs, such as the brain, skeletal muscles, liver, kidney, or placenta muscle [112]. The OPTN functions as a receptor for autophagy after being phosphorylated by TBK1 on serine 177, 473 and 513 [113]. The autophagic adaptor plays a role in several forms of autophagy, including mitophagy, xerophagy, receptor recycling, or aggrephagy, as well as inhibiting the NF-kappa B pathway reducing neuroinflammation [114,115,116]. The inheritance pattern of *OPTN* mutation-associated ALS could be either autosomal dominant or autosomal recessive. However, the disease-related mechanisms can vary between these two traits. For example, a homozygous STOP codon mutation, Q398X, could reduce gene expression by nonsense-mediated decay, whilst the heterozygous mutation, E478G, could result in OPTN-immunoreactive cytoplasmic inclusions [116]. Additional *OPTN* mutations were observed in ALS, including R69L, Q165X, I451T or E516Q [112].

### 3.4. Genes Impacting Cytoskeletal Dynamics or Microtubule Functions

Dynactin 1 (*DCTN1*) encodes the p150Glued protein, a member of the dynactin protein complex. DCTN1 protein directly binds microtubules via the highly conserved N-terminal glycine-rich and cytoskeleton-associated protein (CAP-Gly) domain and plays a role in retrograde vesicle transport. DCTN1 can interact with TDP43 forming co-aggregates in the cytoplasm. Most disease-related mutations of *DCTN1* (such as G71A) are located in the glycine-rich domain [117,118,119]. NMA-related kinase 1 (*NEK1*) is a serine/threonine kinase and a mitotic checkpoint protein, which could control cell cycle progression and mitosis. It could also be involved in DNA repair. *NEK1* was also suggested to regulate the cilia formation, microtubule stability, and dynamics [120]. *NEK1* dysfunctions are associated with brain diseases such as hydrocephalus and mental retardation. In 2016, *NEK1* was initially associated with ALS since a loss-of-function mutation, R261H, was observed as a risk factor for the disease [121]. Additional loss-of-function mutations (such as N181S, G399A, and M545T) were also reported, reducing *NEK1* expression and haploinsufficiency [122].

Profilin-1 (*PFN1*) is a 140 amino acid actin-binding protein that promotes nucleotide exchange on actin, resulting in the conversion of monomeric ADP-actin to ATP-actin [123,124] and the formation of PFN1-ATP-actin complexes. Additionally, *PFN1* binds to phosphoinositides and an extensive network of proteins with poly–L-proline stretches. Some mutations, such as G118V or M114T, could result in disturbances in the polyproline-binding region of *PFN1*, leading to abnormal actin assembly. Another variant, *PFN1* C71G, potentially reduces *PFN1* expression [125], whilst others, such as M114T or E117G, could also result in abnormal autophagic processes resulting in mitochondrial dysfunctions [126].

Peripherin (*PRPH*) is a type III intermediate filament, a multifunctional protein implicated in neuronal processes, cytoskeletal dynamics, axonal progressions, transport and myelination, neurite stability, and growth, with possible roles in processing DNA/RNA, mitochondrial functions, and vesicle transport. In addition, PRPH is considered a risk factor for diseases such as ALS, Charcot–Marie–Tooth Type 2B, diabetes, or enterovirus-A71 infection [127,128]. *PRPH* is overexpressed in injured neurons and promotes axon regeneration. Mutations in *PRPH*, such as R133P and D141Y [129] or splice site variants [130], may form intracellular aggregates promoting neurotoxicity. In addition, the suppression of Neurofilament Heavy Chain (*NEFH*) or Neurofilament Modest Chain (*NEFM*) expression via a small group of ALS-linked miRNAs is shown to perturb cytoskeleton composition and disrupt axon structure and transport exhibiting impaired N-methyl-D-aspartate (NMDA)-mediated calcium influx [131]. 

The Tubulin Alpha 4a *(TUBA4A)* gene is a rare risk gene for FTD and ALS. It encodes a subunit for alpha-tubulin, which forms a heterodimer with beta-tubulin and is involved in microtubule formation [132,133]. Mutations in the *TUBA4A* gene, such as W407X, could disrupt the assembly of alpha and beta tubulins, leading to reduced assembly of microtubules. ALS cases with *TUBA4A* were spinal onset, but upper motor neurons can also be involved. Cognitive dysfunctions and frontal involvement may also be involved [133,134]. Alsin (*ALS2*) is a Rho Guanine Nucleotide Exchange Factor, which could impact young onset autosomal recessive ALS or hereditary spastic paraplegia [135,136]. Mutations in *ALS2* can be either missense, such as I94V, E159K, M368V or affecting RNA splicing, such as IVS7 + 3A>G [137]. The exact pathological role of mutant *ALS2* remains unclear, but *ALS2* may impact cytoskeletal dynamics [135]. It was also suggested that the knockout of *ALS2* could significantly damage postsynaptic subsynaptic reticulum (SSR) functions. *ALS2* defects may also impair late endosome trafficking, synaptic development, and neuroprotection [137].

### 3.5. Genes Involved in the Regulation of DNA/RNA Processing

In addition to *TARDBP* and *FUS*, other genes, such as sentaxin or angiogenin, can potentially influence RNA-related processes. Sentaxin (*SETX*) is a helicase for DNAs and RNAs and is involved in different processes, including DNA repair, replication or recombination, RNA processing, and translation [138]. Several *SETX* mutations (such as N264S, M386T, or T1118I) have been implicated in impaired DNA repair and gene expression [139] associated with autosomal recessive cerebellar ataxia [138] and rare autosomal dominant juvenile ALS.

The TATA box-binding protein (TBP)-associated factor 15 (*TAF15*) protein is closely related to FUS, since they have similar structures and both belong to heterogeneous nuclear ribonucleoparticle (hnRNP) proteins. *TAF15* was verified to regulate RNA synthesis and also protein splicing. Mutations in *TAF15* (A31T, D386N, R388H, or R395Q) may be related to familial and sporadic ALS [140,141]. 

Angiogenin (*ANG*) was suggested as a rare risk factor for Alzheimer’s disease (AD), Parkinson’s disease (PD), and ALS. *ANG* is a ribonuclease A protein which activates ribosomal RNA regulating its transcription. Moreover, its involvement could also extend to angiogenesis, cell survival, and neuronal growth [142]. For example, multiple *ANG* mutants (such as H13R, K40R, K54R V103I, H114R, or R121C) have been associated with impaired RNase activity, leading to a lower rate of cell proliferation and angiogenesis [143].

Polyglutamine (polyQ) expansions in the neuronal stress protein Ataxin 2 (*ATXN2*) gene are reported to cause spinocerebellar ataxia type 2, whereas intermediate (24–34) CAG repeats are considered a risk factor for ALS [144]. Along with *TARDBP*, *ATXN2* may also impact RNA processing control [145]. In addition, animal and cell models revealed that abnormal ATXN2 localization (due to polyQ expansion) increases TDP43 mislocalization to the cytoplasm. Therefore, TDP43 and ATXN2 interaction could be a possible target for ALS drug development [145].

Heterogeneous Nuclear Ribonucleoprotein A1 (*HNRNPA1*) is a member of a heterogeneous nuclear ribonucleoprotein, which could impact mRNA processing, including splicing and RNA assembly, regulating the stability of RNA and gene expression. Mutations in the prion-like domain of *HNRNPA1*, such as P288A, G264R, or D262V, were identified in ALS cases but could also impact other diseases, such as FTD, multisystem proteinopathy (MSP), or inclusion body myopathy (IBM) [146]. In ALS patients, the immunoreactivity of HNRNPA1 was reduced in the motor neurons of the spinal cord. This process may be associated with abnormal RNA processing [147]. TDP43 could regulate the functions of *HNRNPA1*, leading to alternative splicing and aggregation [148,149].

Matrin3 (*MATR3*) is a conserved DNA–RNA binding protein, which may play a role in several mechanisms, such as mRNA stabilization, DNA repair and transport or splicing. Mutations in *MATR3* could impact ALS, FTD, and distal myopathy [150]. Different *MATR3* mutations were identified in ALS, such as S85C, F115C, P154S, or T622A, which were associated with impaired RNA processing, such as abnormal mRNA export, especially the mRNA for *TDP43* or *FUS* [151,152].

### 3.6. The Potential Impact of Genetic Variants on Mitochondrial Functions 

The Coiled-coil-helix-coiled-coil-helix domain containing 10 (*CHCHD10*) mutations, S59L, R15L, or G66V, was suggested to impact FTD and ALS [152,153]. CHCHD10 is a mitochondrial protein spanning the intermembrane space which interacts with optic atrophy 1 (OPA1) and cyclooxygenase enzymes, impacting cristae organization, mitochondrial dynamics, and the stress response [153,154,155]. The *CHCHD10* S59L mutation is thought to evoke dysfunction through gain-of-function mechanisms via irregular oxidative phosphorylation, cristae destabilization, organelle defragmentation, abnormal protein aggregation, and heightened mitochondrial stress response, inhibiting biogenesis and leading to neurodegeneration [156,157,158]. Furthermore, R15L or G66V are also associated with loss-of-function mechanisms, leading to reduced levels of CHCHD10 protein and CHCHD10–Coiled-coil-helix-coiled-coil-helix domain containing 2 (CHCHD2) complexes potentially impairing mitochondrial respiration and promoting haploinsufficiency [158,159,160]. 

The Sigma Non-Opioid Intracellular Receptor 1 (*SIGMAR1*) gene plays a role in neuroprotection, neuroplasticity, and mitochondrial calcium transport through the inositol trisphosphate receptor (IP3) receptor, which impacts both FTD and ALS [161,162]. Thus, associated mutations (E102Q) have the potential to perturb calcium-dependent mitochondrial ATP production, leading to suppressed proteosome activity under high ER stress and lower mitochondrial respiration. Moreover, *SIGMAR1* mutants may contribute to TDP43 mislocalization and ubiquitinoylation, leading to additional mitochondrial and autophagosome impairment in motor neurons [162]. Also, mutant *SIGMAR1* could result in abnormal autophagy and stress granule aggregation. Furthermore, mutant *SIGMAR1* expression may result in abnormal cytoplasmic RNA binding protein assemblies involving TDP43, FUS or MATR3 proteins [163].

### 3.7. Genes, Which Could Impact Other Pathways

Erb-B2 Receptor Tyrosine Kinase 4 (*ERBB*4 or *HER4*) belongs to the tyrosine kinase family and is member of the epidermal growth factor receptor subfamily. ERBB4 could bind to neuregulin proteins (NRGs), leading to ERBB4 activation and elevated tyrosine kinase activity. Activated ERBB4 may be associated with multiple cellular pathways, including neurodevelopment. *ERBB4* gene was established to impact cancers, but it may also play a role in ALS. Mutations, such as R927Q or R1275W, may disrupt the tyrosine kinase activity of ERBB4, resulting in reduced neuregulin downstream signaling. These mutations were associated with slow progressive ALS, where both upper and lower motor neurons were involved, but cognitive function was spared [164,165]. Annexin 11 (*ANXA11*) is a calcium-dependent phospholipid-binding protein that could impact ALS and FTD onset. Mutations in *ANXA11*, such as D40G, G228Lfs*29, H390P, or R456H, could be associated with both gain- and loss-of-function mechanisms, including abnormal protein aggregation and loss of intracellular calcium responses, respectively. Also, the mutations may result in abnormal stress granule dynamics. ANXA11 protein could co-aggregate with other ALS-related RNA-binding proteins, such as FUS or hnRNPA1, inside lower motor neurons or the brain [166,167,168]. Calcium-Responsive Transactivator (*CREST* or *SS18L1*) is a calcium-dependent transcription activator involved in dendrite growth. It was established to impact cancer onset, but its role in ALS is also investigated. The exact mechanisms of how *CREST* mutations may be related to ALS remained unclear. Mouse studies revealed that an ALS-related *CREST* mutation, Q388X, could repress the cytokine expression (C-C Motif Chemokine Ligand 2; Ccl2 or C-X-C motif chemokine ligand 10; Cxcl10) inside neurons [169]. D-Amino Acid Oxidase (*DAO*) could cause ALS and bipolar diseases. DAO protein regulates brain D-serine, which could act as a neuromodulating factor. Also, DAO may have a neuroprotective role in the brain by protecting against D-amino acid aggregation by modulating their degradation. Furthermore, DAO may also impact dopamine synthesis. ALS-related *DAO* mutations like R199W may be associated with elevated D-serine levels. The D-serine could accumulate in the spinal cord, leading to motor neuron degradation [170,171]. 

### 3.8. Copy Number Variants (CNVs) in ALS

Besides the repeat expansions in *c9orf72*, *ATXN2*, and *NIPA1*, the copy number variants (CNVs) are associated with the gain (multiplication) or loss (deletion) of a certain sequence into the genome, which could result in abnormal protein expression. The CNVs were suggested to impact different disorders, including CMT, autism, schizophrenia, and epilepsy, but they could also play a role in ALS [172]. For example, the survival motor neuron (*SMNs*, *SMN1* and *SMN2*). SMN proteins may impact several cellular functions, including RNA splicing, recycling, and axonal transport. SMN gene mutations could impact motor diseases, including spinal muscular atrophy (SMA). *SMN1* duplications were suggested to play a role in sporadic ALS, but they failed to find an association between *SMN2* and ALS. However, further studies are needed on the mechanism of *SMN1* duplication in ALS onset [173,174]. A study by Moisse et al. (2021) failed to find an association between ALS risk and *SMN1* or *SMN2.* Also, this study suggested that SMN genes may not impact the ALS disease course or disease severity [175]. Blauw et al. (2008) investigated studies on CNVs, which may be a risk for ALS, but they did not find any common CNVs in ALS patients. However, they found gene deletions (including Crystallin Beta B3-*CRYBB3*, Crystallin Beta B2-*CRYBB2*, LDL Receptor Related Protein 5 Like-*LRP5L*, Crystallin Beta B2 Pseudogene 1-*CRYBB2P1*), which were more common in ALS patients, compared to controls, suggesting CNVs as possibly ALS susceptibility factors [176]. An additional study by the same group performed a large genome study on CNVs and suggested that dipeptidyl-peptidase 6 (*DPP6*) and *NIPA1* CNVs may be possibly promising candidates for ALS association [177].

## 4. Potential ALS Biomarkers 

ALS is a complex disease that shares similar phenotypes with other diseases, such as FTD or PD. To limit misdiagnosis, a battery of comprehensive tests, including brain-and spine imaging, biofluid analysis, and neuropsychological tests, are routinely employed [74]. Current ALS diagnosis utilizes the simplified Gold Coast criteria (evolved from the revised El Escorial and the Awaji criteria), which employs three stages. The first stage involves progressive motor impairments documented from repeated clinical assessments preceded by normal motor function. The second stage involves the identification of upper (UMN) and lower motor neuron (LMN) dysfunctions in at least one of the four body regions (bulbar, cervical, thoracic, and lumbar) or LMN dysfunction in at least two regions via electromyography (EMG). The final stage is finding evidence to exclude other disorders. Electromyography (EMG) remains an unrivalled diagnostic tool, but it is far from 100% sensitive, resulting in significant diagnostic delays regarding motor neuron disease (MND). As a supplement or alternative to EMG, several molecular and imaging biomarkers can be employed to monitor MND progression and assess the efficacy of potential therapeutic candidates [178,179]. Proteomic markers may be the easiest to apply, as they are compatible with current methods such as traditional mass spectrometry, immunoassays, or aptamer technologies. Moreover, protein profiling is compatible with various biofluids such as plasma, serum or cerebrospinal fluid (CSF) [179,180].

Given the diversity of pathological mechanisms related to ALS (excitotoxicity, oxidative stress, neuroinflammation, metabolism dysfunctions) [178], a biomarker panel must rule out the possibility of other diseases that closely mimic ALS (spinal and bulbar muscular atrophy (SBMA) and multifocal motor neuropathy (MMN)), allow the disease risk prediction, identify patients genetically suited for trials, reduce the time of diagnosis, and provide the earliest opportunity to start therapy [181]. 

A novel set of ALS-specific biomarkers might include a combination of multiple biomarkers, including neurophysiological and neuroimaging and markers from biological fluids and tissues, which could effectively diagnose and monitor the disease progression [182]. Altered gene expression analysis after muscle biopsy may help distinguish the earlier and later forms of disease pathology. However, a biopsy is an invasive method that is not always required for disease diagnosis [183], whereas fluid biomarkers (neurofilaments (NFs) sourced from Table 3, CSF, serum, or blood [184,185,186,187,188,189,190,191,192,193,194,195,196,197,198,199,200,201,202,203,204,205,206,207,208,209,210,211,212,213]) associated with ALS-related changes in the brain [182] can provide a less invasive option. 

### 4.1. Biomarkers of Disease Diagnosis and Differential Diagnosis

Neurofilaments are part of the cytoskeleton; their level could be higher in biological fluids in case of axonal damage. NFs have three subunits: light (NFLs), medium (NFMs), and heavy (NFHs) chains. It was suggested that their fluid levels potentially reflect the degree of axonal injury in the case of neurodegenerative diseases [183]. Multiple studies revealed that NFs in blood or CSF were elevated in different neurodegenerative diseases, such as ALS, parkinsonism, AD, or multiple sclerosis [184,185,186,187]. NFLs have been extensively studied in ALS. For example, Verde et al. (2019) revealed that serum NFLs in ALS patients were higher than in controls and non-ALS patients (AD, FTD, or PD), aiding differential diagnosis. A recent study (*n* = 429) further highlighted the potential of serum NFL to distinguish ALS from other diseases, implying that the routine examination of serum NFL can improve survival prediction and diagnostic accuracy [188]. In addition, Sun et al. (2020) measured NFL in CSF, and sporadic ALS patients revealed higher levels of NFLs compared to controls. Unfortunately, this study failed to find significant differences in CSF NFL levels between ALS and other CNS or peripheral neuropathy diseases. These findings suggested that CSF-NFL levels cannot significantly distinguish ALS from other diseases. However, a negative correlation was found between ALS function scores and CSF-NFL levels, implying CSF-NFL might be a helpful marker in monitoring disease severity and progression [184,189]. Besides NFL, phosphorylated NFH (pNFH), present in (CSF) and serum, offers similar diagnostic utility comparable to NFL. In a recent study, including ALS patients (*n* = 234), ALS mimics (*n* = 44), as well as non-neurological disorder controls (*n* = 9), SF NFL, CSF pNFH, and plasma NFL levels were significantly increased in ALS patients, with authors concluding pNFH had diagnostic value capable of differentiating ALS from clinically relevant mimics [190]. Higher levels of pNFH in serum and CSF may also correlate with the degree of motor neuron impairment [191]. In addition, NFHs in plasma may also reflect the degree and pace of neurodegeneration, disease progression, and survival [192]. Behzadi et al. (2021) suggested that neurofilaments may be higher in ALS patients than in patients with other motor neuron diseases (including spinobulbar muscular atrophy, progressive muscular atrophy), myopathies, or neuropathies. Furthermore, neurofilaments may be able to distinguish the different ALS subgroups (spinal-bulbar or truncal onset). Bulbar-onset ALS patients presented higher levels of plasma NFLs than spinal-onset patients, but there was no difference in patients with truncal-onset. No difference was seen in the CSF-NFL levels among the different subtypes [190].

Multiple studies have confirmed that inflammatory cytokines and T-cell-related immune response are elevated in body fluids of ALS patients, compared to unaffected age-and gender-matched controls [193,194,195,196], suggesting the disease might have a unique inflammatory pattern. Cytokines in plasma may also be useful in ALS diagnosis. For example, Tortelli et al. (2020) analyzed five inflammatory markers (interleukins, such as IL-2, IL-6, IL-10, interferon; IFN-gamma, and tumor necrosis factor; TNF-alpha) in plasma; all were higher in patients compared to the unaffected controls, with IL6 being the most potent marker [197]. Whether inflammation is causal, exacerbates disease pathogenesis, or is the consequence of neurodegeneration is debatable. Also, further research is needed on whether the inflammatory response could be damaging or protective [193,197]. In 2017, a serum analysis of ALS patients showed elevations for malondialdehyde (MDA), 8-hydroxy-2′-deoxyguanosine (8-OHdG), the ratio of glutathione disulfide (GSSG)/glutathione (GSH), and IL6 and IL8, indicating a reduction in the total antioxidant status (TAS) of ALS patients [198]. Additional serum analysis involving 106 serum cytokines, growth factors, and markers for blood–brain barrier breakdown, sourced from 15 patients, was conducted by Cao et al. (2022). No significant differences were detected, with authors attributing the findings to small sample size; however, 20 markers involved in angiogenesis and growth were reportedly enriched (such as fractalkine, Brain-derived neurotrophic factor BDNF, Epidermal Growth Factor EGF, Platelet-derived growth factor PDGF, Dickkopf WNT signaling pathway inhibitor 1, macrophage migration inhibitory factor, angiopoietin-2, or S100 calcium-binding protein B) and segregated with ALS [199].

### 4.2. Biomarkers of ALS Prognosis and Disease Progression

Early studies showed CSF-Tau could be a reliable marker for several neurogenerative diseases, including ALS [200,201]. However, its role regarding ALS may be limited [200], as Scarafino et al. (2018) [201] and Kojima et al. (2021) [202] highlighted that its inclusion was best served as a disease prognosticator. More recently, Thapa et al. (2023), via a meta-analysis of ALS studies, demonstrated that the t-Tau to pTau/tTau ratio was a better indicator of ALS in patients than either alone [203]. In addition, Sun et al. (2020) monitored eight prognostic markers (including serum creatine, albumin, C-Reactive Protein CRP, and glucose, calcium, and potassium) in the blood of 399 ALS patients. The study highlighted an association between disease biomarkers and the possible risk of higher mortality. In conclusion, the authors suggested further studies were needed regarding the potential application of these markers in ALS prognosis [204]. Other studies have shown that ALS patients with c9orf72 repeat expansion tend to have higher pNFH levels in CSF, which can exhibit quicker disease progression and shorter survival times than those without c9orf72 expansion. Such findings suggest that pNFH may be helpful in the prognosis of disease progression with or without intervention in patients with c9orf72 expansion [205].

Gaiani et al. (2017) investigated a study on NFL levels in the blood and CSF of ALS patients. This study revealed that NFLs can potentially support disease diagnosis and prognosis in ALS patients. High early-stage NFL levels may be associated with poorer disease outcomes [206]. Vacchiano et al. (2021) measured NFL in plasma and CSF using the SIMOA platform to differentiate ALS patients from those who mimic ALS. NFL concentrations were higher in plasma and CSF than those with ALS mimic. ALS patents’ quick disease progression and shorter survival showed higher NFL levels in both plasma and CSF [189].

TDP43 is a neurodegenerative marker frequently used to confirm AD and FTD [207,208] and, more recently, as a potential candidate for ALS progression. For example, Beyer et al. (2021) revealed that the beta-sheets of TDP43 sourced from the CSF of ALS patients were significantly more enriched compared to TDP43 samples taken from PD patients and controls. In support of those observations, faster progressive ALS patients have also exhibited higher beta-sheet enrichment than slower progressive patients [209].

Other inflammatory markers in a potential ALS panel are the metalloproteases (MMPs) MMP-2 and MMP-9, and their tissue inhibitors (TIMPs), essential in regulating enzyme activity. TIMPs, specifically TIMP-1, could inhibit the proteinase activity of several MMPs, impacting ALS progression [210,211,212]. For example, early studies associated higher MMP9 levels with peripheral nerve and muscle damage, a common feature in the disease’s initial stage [213]. A membrane type-matrix metalloproteinase (MT-MMP1) and MMP9 in serum could be potential markers in distinguishing ALS patients from asymptomatic individuals [221].

In another study, using larger sample (CSF and serum) sizes (*n* = 105), researchers evaluated the potential of secreted macrophage and epithelial proteins, Chitotriosidase-1 (CHIT1), chitinase-3-like protein 1 (YKL-40), and monocyte chemoattractant protein-1 (MCP-1) as ALS prognostic biomarkers. CSF levels of these markers were elevated in ALS patients compared to the controls; however, they may not be effective as discriminatory markers between ALS and ALS mimics. A correlation was observed between CHIT1, YKL-40, and disease progression rate. The CSF CHIT1 levels were higher in the ALS patients, who presented more regions with motor neuron degeneration. The YKL-40 and MCP-1 in CSF could predict the disease survival of ALS patients. Furthermore, CHIT1 and YKL40 were weakly correlated with disease progression rate [210]. Other chitinases, such as chitinase-3-like-2 (CHI3L2) or chitinase-3-like-1 (CHI3L1), were also elevated in the CSF of ALS patients, compared to controls. However, they may not help distinguish ALS from primary lateral or multiple sclerosis. Chitinase levels in ALS patients may correlate with molecules involved in neuroinflammation, such as MCP-1, C-reactive proteins or soluble TREM2. Also, CHIT1 expression may correlate with interleukin expression (IL18 or IL16), and CHI3L1 and CHI3L2 may correlate with complement molecules, such as C1 components. These findings suggested that chitinases may be markers for inflammatory processes in ALS [219,220,223]. Perivascular fibroblast proteins, such as secreted phosphoprotein 1 (SPP1) and Collagen Type VI Alpha 1 Chain (COL6A1), were analyzed in the plasma of European (Swedish, Dutch, German, and Belgian) ALS patients and controls. High SPP1 and COL6A1 were associated with shorter survival [222].

### 4.3. Biomarkers of ALS Onset Prediction

Predicting the impairment of regulatory processes and the formation of abnormal DPR proteins [224] due to enlarged C9orf72 repeat expansions may be one of the risk factors for ALS. Thus, elevated c9orf72 poly-GP protein levels in clinical (CSF and peripheral blood mononuclear) samples could be prognostic for *c9orf72* carriers. Elevated levels of poly-GR and poly-GPs can appear in patients and asymptomatic carriers, and are reduced in murine models following exposure to antisense oligonucleotides (ASO) [215], suggesting the protein has potential in assessing the rescue effects of therapeutic candidates. In addition to poly-GP, elevated polyGR, polyGA, and *SOD1* have exhibited [216] similar potential following ASO treatments [217,218]. Abnormal TDP43 splicing (with cryptic exon inclusion) is thought to occur before disease onset, suggesting that alternatively spliced TDP43 can potentially predict ALS onset [214]. NFL may also be useful in identifying pre-symptomatic individuals. Benatar et al. (2020) revealed that serum and CSF NfL levels of asymptomatic carriers of fALS mutations were higher than the controls but lower than the ALS patients. Elevation of NFL levels may predict ALS onset approximately a year before the clinical symptoms appear [225]. The CHIT1 elevation appears to be a feature of the late pre-symptomatic to early symptomatic phases in patients carrying mutations in C9orf72 or SOD1 [219].

### 4.4. MiRNAs and Extracellular Vesicles in ALS

The aberrant expression of microRNAs (miRNAs) and extracellular vesicle contents are potential indicators of neurodegeneration and disease progression [226,227,228,229,230]. For example, miRNA abnormal expression has been associated with suppressed mRNA levels in spinal motor neurons and the peripheral white blood cells of ALS and SALS patients (Table 4). In particular, the study involving SALS patients demonstrated that four miRNAs (hsa-miR-34a, hsa-miR-100, hsa-miR-193b, and hsa-miR-4485) were up-regulated, and seven miRNAs (hsa-miR-3690, hsa-miR-124, hsa-miR-183, hsa-miR-3935, hsa-miR-451, hsa-miR-4538, and hsa-miR-4701) were down-regulated in patients compared to controls. The authors concluded that five miRNAs (hsa-miR-183, hsa-miR-193b, hsa-miR-451, and hsa-miR-3935) showed potential as leukocyte markers for ALS, whilst the underexpression of hsa-miR-183 in SALS warranted further investigation [231,232,233]. Another miRNA for consideration is miR-338-3p, present in the blood, serum, and CSF fluids. The higher sample quality of CSF (due to the absence of high-density lipoproteins) renders it the most sensitive to pathological changes (synaptic dysfunction and neuronal loss) in the initial and progressive stages of sALS. Early investigations showed miR-338-3p elevations are present in all three sample fluids sourced from SALS patients. Moreover, miR-338-3p expression was up-regulated in the grey matter of the spinal cord [234], suggesting the marker could provide additional insight into SALS pathogeneses. In another study, up-regulated miR181a-5p and down-regulated miR21-5p and miR15b-5p were observed in CSF. Additionally, the ratio of these markers (miR181a-5p/miR21-5p and miR181a-5p/miR15b-5p) presented high sensitivity and specificity for ALS [226].

The potential outlook regarding the clinical application of miRNA signatures for SALS is somewhat limited, given the lack of multi-center studies and serum miRNAs. In an attempt to address this issue, a recent study examined miR-181 levels in 252 ALS patients and found an association between higher levels in the plasma and a higher risk of mortality. Moreover, when miR-181 was paired with NFLs, the combination offered superior prognostication capacity for ALS, leading to improved patient stratification [235].

Other potential serum biomarkers include miR-206, 143-3p, and 374b-5p, which may also improve ALS diagnosis and prognosis. For example, the expression of miR-206 and miR-143-3p was higher in ALS patients, and miR-374b-5p expression decreased compared to controls, suggesting miRNAs may have a role in monitoring patients [236]. In addition, several other miRNAs were up-regulated (including miR-124-3p [237], miR-151a-5p, miR-146a-5p [238]) or down-regulated (for example, miR-4454, miR-10b-5p, miR-29b-3p [238]) in the extracellular vehicles (EVs) of ALS patients, suggesting miRNA sourced from EVs may be promising candidates for ALS diagnosis and prognosis [237,238,239].

**Table 4:** Examples of miRNAs as markers for AD diagnosis/prognosis.

miRNA	Subjects	Biofluids/Tissues	Results	Reference
miR-146a, miR-524-5p and miR-582-3p	3 controls and 5 sALS	spinal cord lysates	These miRNAs were down-regulated in ALS, regulating the NFL mRNA	[232]
hsa-miR-183, hsa-miR-193b, hsa-miR-451, and hsa-miR-3935, etc.	5 controls and 5 sALS, validation: 83 SALS and 61 controls	leukocytes	hsa-miR-183, hsa-miR-193b, hsa-miR-451, and hsa-miR-3935 were down-regulated in ALS patients. The hsa-miR-183, hsa-miR-193b, hsa-miR-451, and hsa-miR-3935 may be useful in sALS diagnosis	[233]
miR-338-3p	72 sALS, 62 controls (leukocytes), 10 controls, 10 sALS (CSF), 7 sALS, 3 control (spinal cord)	Leukocytes, CSF, serum, spinal cord	miR-338-3p was up-regulated in leukocytes, CSF, serum, and spinal cord of sALS patients miR-388-3p was localized in the grey matter of ALS patients	[234]
Several miRNAs	24 ALS, 24 controls	CSF	miR181a-5p was up-regulated, miR21-5p and miR15b-5p were down-regulated miR181a-5p/miR21-5p and miR181a-5p/miR15b-5p ratios may be useful in ALS detection	[226]
Circulating miR-181	252 ALS patients	plasma	miR-181 could be a possible prognostic marker	[235]
miR-206, miR-143-3p, miR-374b-5p	27 sALS, 25 controls	Serum	miR-206 and miR-143-3p were increased and miR-374b-5p was decreased in patients.	[236]
EV miRNAs	50 patients, 50 controls	Plasma EVs	hsa-miR-4454, miR-10b-5p, miR-29b-3p were down-regulated, miR-151a-5p, miR-146a-5p, miR-199a-3p, miR-199a-5p, miR-151a-3p were up-regulated in ALS patients, possible diagnostic candidates	[237]
	6 sALS, 9 FTD, 6 AD, 9 PD, and 9 controls	Plasma small and large EVs	Deregulated miRNAs in EVs of ALS patients (such as hsa-miR-206, hsa-miR-205-5p, miR-1-3p, hsa-miR-205-5p), EVs may be helpful in differential diagnosis	[238]

The contents of EVs can potentially be employed to diagnose different stages of ALS and other neurodegenerative diseases. The vehicles are involved in the transport and exchange of RNAs, lipids, and proteins between recipient cells. Since they can cross the BBB, they may accurately reflect the disease-specific changes inside the brain. Also, they may be sufficiently present in peripheral areas, making them easy to obtain from several sources, including plasma, whole blood, CSF, or saliva. Alterations in the protein/RNA contents were suggested as promising markers that may reflect the disease-related changes [237,238,239,240,241].

Regarding storage, EVs have a double membrane, which could preserve and protect RNAs and protein content [240,241,242,243,244,245], different proteomic markers were detected from EVs sourced from patients at different stages of the disease. For example, TD43 protein (c-terminal fragments and full-length protein) was elevated in the CSF of ALS and ALS/FTD patients compared to unaffected individuals [244]. Other CSF proteins were also altered in sporadic ALS patients, compared to non-ALS controls (idiopathic normal-pressure hydrocephalus). Hayashi et al. (2020) performed protein analysis from exosome-enriched CSF fractions. They reported elevated INHAT repressor (NIR), nucleolar complex protein 2 homolog (NOC2L), programmed cell death 6-interacting protein (PDCD6IP), versican core protein (VCAN) proteins, and 11 proteins, which were reduced in ALS patients (such as alpha-1-antichymotrypsin, SERPINA3; receptor-type tyrosine-protein phosphatase zeta, PTPRZ1; complement C1q subcomponent subunit C, C1QC; coiled-coil domain-containing protein 19, mitochondrial, CCDC190), with researchers proposing that nucleolar stress impairment may impact sporadic ALS [233].

Elevated TDP43 and hyperphosphorylated TDP43 levels have been reported in plasma EVs [242,243]. TDP43 in EVs may also be a valuable biomarker to monitor ALS progression since their contents can vary with disease progression [243]. Pasetto et al. revealed reduced levels of Hsp90 in plasma EVs compared to controls [244]. Immune molecules, such as IL6 (systemic pro-inflammatory), were also elevated in plasma EVs, which may reflect the presence of neuroinflammation in the brain of ALS patients [246].

## 5. Discussion and Future Promise of ALS Therapy

ALS is a motor neuron disease which could affect the neurons responsible for muscle movement control. The phenotypes of ALS may overlap with other disorders, including FTD or PD, which may make the diagnosis more difficult [247]. ALS is a genetically complex disease since multiple genes were suggested as causative or risk factors. Besides *SOD1*, *c9orf72*, *TARDBP*, and *FUS*, many other rare disease-causing genes were discovered, which may impact either familial or sporadic forms of ALS. With the availability of next-generation sequencing technologies, the cost and time of sequencing has been reduced. With novel sequencing technologies, more and more novel genes were discovered, not only in the coding but also in the non-coding regions. The discovery of new genetic variants revealed that multiple pathways might be involved in ALS progression, including regulation of gene expression, protein trafficking, or autophagy [10,18]. Recently, the discovery of ALS-related genetic factors has been accelerated, and in the future, more possible candidates may be discovered [248,249]. Understanding the involvement of different genetic factors in ALS progression is an excellent challenge in disease-related research. Determining the possible disease-causing, risk factor genes or other biomolecules is becoming increasingly important in disease diagnosis or therapy [249,250]. In the last few years, multiple biomarkers and possible candidates for ALS were identified in biological fluids, such as CSF or blood. The discovery of biomarkers should be essential in disease diagnosis, monitoring its progression, and screening the efficacy of drug candidates. Research on potential biomarker candidates is ongoing, and several promising proteomic markers were discovered, including NFLs, Tau protein, CHIT1, and other inflammatory markers [250].

MiRNAs have multiple roles, from regulating gene expression (mRNAs inhibition) to impacting several neurological processes, including neurodevelopment, neuroprotection, or maturation of brain cells. Impairment in miRNA functions may be associated with neurodegenerative processes, including dysfunctions of motor neurons. Dysregulation of miRNAs may be a common hallmark in ALS. Several miRNAs were dysregulated in ALS (such as miR21-5p, miR-206, miR15b-5p, or miR-338-3p), but further studies are needed to understand how the miRNAs could impact ALS diagnosis, prognosis and therapies [214,217,218,225,226,227,228,229,230,231,232,233,234,235,251,252]. Screening proteins and miRNAs in EVs may also improve the disease diagnosis [237,238,239,240,241,242,243,244,245,246].

ALS is clinically recognized as a heterogenic (upper or lower motor neurons, or both affected, bulbar or limb onset) disease with a complex molecular background. As mentioned previously, novel genetic factors continue to be identified, whose functional role in ALS involving endosome composition, autophagy, or cytoskeletal system is yet to be determined. In addition, several biomarkers (proteomic markers or miRNAs) in the biological fluids could play a role in ALS diagnosis, prognosis, or prediction (either the clinical course or the disease onset). Moreover, these markers’ timely presence may indicate a patient’s responses to therapies [253,254]. As such several novel methods (such as combined tissue–fluid proteomics) were developed to discover possible biomarker candidates, some with applications in phenotypic stratification [255]. ALS stratification is essential for optimized personal clinical care and treatment. For example, phenotypic studies involving relatively small cohorts suggest neurofilament markers could be used in ALS patient stratification [256]. With the aid of machine learning and a novel scheme, ”The Stratification Challenge” (DREAM Prize4Life ALS), Kueffner et al. (2019) successfully performed clustered analysis and the classification of ALS patients and ALS subgroups [257].

Similar to most neurodegenerative diseases, no successful therapy is currently available for ALS. However, promising strategies are available through gene therapy. In other motor neuron diseases, spinal muscular atrophy (SMA), and several gene therapeutic approaches, such as Nusinersen, Zolgensma, or Risdiplam, have been approved, which could lead to improved life quality of patients. The gene therapy strategy may also be promising for ALS drug development [258,259]. Antisense molecule delivery, which targets genetic mutations in *SOD1*, *FUS*, *TARDBP*, or *c9orf72*, was suggested to be helpful in disease therapy. The gene therapy methods may be helpful in patients positive for ALS-causing mutations. For example, ALS patients who received ASO-Tofersen were associated with reduced SOD1 and NFL levels in CSF and plasma of ALS patients, respectively. However, the drug did not improve the clinical symptoms of patients [260]. Tofersen or BIIB067 drug may be helpful to SOD1 carriers in pre-symptomatic ALS individuals with higher levels of plasma NfL. The drug may delay the disease’s progression and is currently in phase III clinical trials [261]. Another potentially available ASO for *c9orf72* repeat expansion is *C9orf72*-631 or BIIB078. BIIB078 may protect the neurons from gain-of-function toxicity of repeat expansion and may prevent glutamate-related toxicity, and is in phase I of clinical trials [262,263,264]. For *FUS* mutations, an ASO-related drug called Jacifusen (NCT04768972 or ION363) is also in phase III of clinical trials [265].

Research is ongoing on viral-based strategies of gene therapy as well. For example, adeno-associated virus (AAV)-related gene therapies may also be promising approaches in ALS mutants. AAV-related gene silencing in *SOD1* mutant mice showed reduced SOD1 expression and improved motor skills [266]. AAV-miR-*SOD1* was tested in two ALS patients with *SOD1* mutants. A slight improvement was observed in one of the patients in leg strength, while the other patient’s condition remained stable. These findings suggested that AAV-related gene silencing may slow down the disease progression. However, AAV therapy may need immunosuppression to avoid side effects, such as meningoradiculitis [267,268]. AAV-related gene silencing was also attempted to target *c9orf72* repeat expansion in ALS mouse models (AAV5-miC). These mice showed reduced RNA aggregation with repeat expansion in nerve cells, leading to reduced neurotoxicity [269]. The AAV5-related gene silencing was also screened in human cell lines, such as HEK293T- and iPSC-derived neurons, and it showed reduced levels of mutant *c9orf72* mRNA in both plasma and cytoplasm of the cells. In addition, RNA foci were reduced in the nucleus [270]. Studies are going on to “reverse the mutation” by genome editing (such as the CRISPR-Cas9 method) for *SOD1*, *C9orf72*, *FUS*, *ATXN2*, and *TARDP* mutations. These studies showed promising data in iPSC cell lines derived from ALS patients, such as increased survival and reduced mutant protein-related toxicity [268].

Treatment of neurodegenerative diseases may be more effective in the early disease stage or even in the pre-symptomatic phase. Studies are needed regarding pre-symptomatic ALS to identify the individuals who may develop disease phenotypes. Genetic analysis may effectively identify at-risk individuals; however, the known genetic factors (such as *SOD1*, *c9orf72*) may not define all ALS cases. Several biomarkers, such as elevated NFL in the serum and CSF, may be present years before the disease symptoms appear. As well as the lack of definitive markers or ratios, pre-symptomatic ALS diagnoses face many other challenges. One of the most significant is a patient’s psychological readiness before pre-symptomatic testing, comprehension of a positive result, their legal protections regarding information disclosure, and potential discrimination based on genetic results, particularly if a mutation or risk factor is present; counseling should be tailored. The other issue with pre-ALS testing is the logistical complexity. For example, potential pre-ALS individuals may live remotely and are inexperienced with telemedicine. Moreover, if trained personnel are unavailable, monitoring the initial stages of the disease may go undetected, particularly given the overlap of FTD with ALS, leading to further complications [271,272,273,274].

Even though studies have yielded promising results, research on gene therapies may have limitations. One difference between SMA and ALS is that most SMA cases (type I and II) occur in early childhood, but most ALS cases are adult onset. Therefore, the gene therapies for SMA may be given before the second year of age, but the treatment may not be efficient later, especially after the clinical symptoms appear [240]. The other issue with therapy is that ALS is a genetically complex disease; gene therapies may not be available yet for all ALS-related genetic factors. Nevertheless, the gene therapies may work in the pre-symptomatic disease stage, suggesting that obtaining genetic and biomarker data may be essential to identify the “at risk” individuals. Discovering the early markers in pre-symptomatic individuals may also improve ALS drug development [261,271].

## 6. Conclusions

ALS is a complex disease, since many diseases associated genes were identified, including SOD1, c9orf72, TARDBP, or FUS. Emerging studies are also discovering new genetic factors, which could cause disease or contribute to disease progression. Also, several proteomic (including NFs, Tau, TDP43, inflammatory) and microRNAs were discovered, which could play a role in disease diagnosis, prognosis, and prediction. Diagnosing ALS in early or even pre-clinical stages should be important. Currently, there are no therapies available for ALS. However, gene therapies were successfully adapted against other motor diseases, such as SMA. Also, ASO-based gene therapies (such as Tofersen or Jacifusen) are currently in clinical trial phases, but AAV-based therapies are also under development. Finding the possible genetic and proteomic markers for ALS diagnosis, prognosis, and prediction should be essential for gene therapies against the disease.

## Figures and Tables

**Table 1:** ALS involvement and significant mutations in *SOD*1, *C9orf72*, *TARDBP* and *FUS* genes [18].

Gene	Function	Pathways, ALS Impact	Examples of Significant Mutations	Additional Disease Impact
*SOD1*	Cu–Zn superoxide dismutase, protection against oxidative stress, proteosome turnover	Abnormal SOD1 dimerization, aggregation, proteosome dysfunctions Calcium homeostasis is impaired, neuroinflammation	A4V H46R, G93R	Spastic Tetraplegia, Axial Hypotonia, Progressive
*C9orf72*	Regulation of gene expression, splicing, endosome function and autophagy	Abnormal splicing, haploinsufficiency, DPR aggregates, reduced gene expression	G4C2 repeat expansion (over 30 possibly pathogenic)	FTD (possible association with AD and PD)
*TARDBP*	Regulates the splicing, transport of RNA and miRNA generation	TDP43 aggregation, TDP43 mislocalization, abnormal protein interaction Possible propagation	Q331K, M337V, Q343R, N345K, R361S, N390D	FTD
*FUS*	Regulates the splicing, transport of RNA and miRNA processing, impacts the genome integrity	FUS overexpression, FUS aggregation, nonsense-mediated decay Possible propagation	R521 mutations, R495X, R522G, ΔExon15	FTD

**Table 2:** Examples of rare genetic factors and their potential roles in ALS (“fs” represents frameshift mutation) [18].

Gene	Function	Pathways, Impact ALS	Examples of ALS-Related Mutations	Additional Disease Impact
*CHMP2B*	Sorting endosomes and forming endo-lysosomes	Abnormal TD43 phosphorylation by slower ubiquitination of CK3	Q165X, Q206H	FTD, HSP
*VCP*	Chaperon functions, autophagy, protein QC, vesicle trafficking	TDP43 mislocalization, abnormal autophagy	R159H, I114V	IBM, Paget’s disease, FTD
*CCNF*	E3 ubiquitin-protein ligase	Impairing the proteosome turnover, disturbed E3 ligase activity	S621G, S195R, K97R, S509P, I772T, S3G, K97R	FTD
*UBQLN2*	Ubiquitin proteosome system, protein QC	Reduced proteosome-mediated degradation, impaired autophagy, neuroinflammation	R497H, P506S, P509S	FTD
*HSB1*	Molecular chaperon, proteosome maintenance	Loss of chaperon, impaired proteosome	Q190H, A204Gfs* 6	CMTD, MND
*VAPB*	Vesicle trafficking, formation of neuromuscular junctions, Ca homoeostasis	Disorganization of ER structures	P56S	NA
*NIPA1*	Magnesium transporter, early endosome regulation	Abnormal splicing, haploinsufficiency, reduced gene expression	GCG repeat expansion	Prader-Willi/Angelman syndrome 1
*SPG11*	Vesicle transport	lysosome trafficking, cholesterol transport, autophagy, calcium homeostasis	c.704_705delAT, c.5199delA	HSP
*FIG4*	Vesicle transport in the endosome/lysosome system	Abnormal endosome process	I41T, F254Sfs*8, Y647C	CMT, motor diseases
*SQSTM1*	p62 in protein degradation, aggregation, autophagy	oxidative stress, abnormal autophagy	L341V, G427R	FTD
*TBK1*	NF-κB activator, mitophagy, autophagy, autoimmunity	Abnormal auto-and mitophagy, reduced autophosphorylation, autoimmunity, inflammation	D135N, G217R, R357Q, M559R	FTD
*OPTN*	Different forms of autophagy, protection against inflammation	Reduced gene expression (stop codon mutations), OPTN immunoreactions, inflammation	Q398X, E478G, R69L, Q165X, I451T, E516Q	glaucoma
*DCTN1*	Binding of microtubules, vesicle transport, DNA repair	Abnormal vesicle and microtubule functions	M571T, R785W, R1101K	FTD, MNDs
*NEK1*	Mitotic checkpoint, microtubule and cilia functions, DNA repair	Reduced NEK1 expression, actin impairment	R261H, N181S, G399A, M545T	NA
*PFN1*	Regulation of actin functions, cytoskeleton functions	Abnormal actin assembly, impaired autophagy, mitochondrial dysfunctions	G118V, M114T, C71G	MNDs, such as HD
*PRPH*	Different cytoskeletal processes, DNA-RNA processing, vesicle transport, axon regeneration	Cytoskeletal dysfunctions, formation of intracellular aggregates	R133P, D141Y	CMT, diabetes enterovirus-A71 infection
*NEF genes*	Regulating cytoskeleton formation, axon dendrite transport	Reduced expression, cytoskeletal dysfunctions	S586F, K599T	CMT
*TUB4A*	Cytoskeleton regulation, microtubule formation	Reduced microtubule assembly	W407X	FTD
*ALS2*	Rho Guanine Nucleotide Exchange Factor	Multiple impairments, such as cytoskeleton issues, SSR dysfunctions	I94V, E159K, M368V, IVS7 + 3A > G	HSP
*SETX*	Helicase regulates the gene expression and DNA/RNA repair	impaired DNA repair and gene expression	N264S, M386T T1118I	ataxia
*TAF15*	RNA synthesis, protein splicing	Impaired synthesis of RNA, RNA aggregation	A31T, D386N, R388H, R395Q	Leukemia, cancer
*ATXN2*	Regulation of RNA processing with TARDBP	Enhancing the TDP43 toxicity	PolyQ repeats	SCA2, FTD
*HNRNPA1*	mRNA processing, splicing, gene expression	Impaired RNA regulation processes	P288A, G264R or D262V	FTD, MSP, IBM
*MATR3*	Regulation of gene expression, DNA repair	Impaired RNA processing, abnormal TDP43 and FUS transport	S85C, F115C, P154S, T622A	FTD, distal myopathy
*ANG*	Activates the ribosomal RNA, regulates the transcription and ribosomal RNA synthesis, angiogenesis	Reduced RNAse activity, reduced cell proliferation and angiogenesis	H13R, K40R, K54R V103I, H114R, R121C	PD, AD
*CHCHD10*	Regulates mitochondrial dynamics and its response to stress	Mitochondrial fragmentation, cristae disorganization Haploinsufficiency	S59L, R15L, G66V	FTD
*SIGMAR1*	neuroprotection, neuroplasticity, mitochondrial Ca transport	Reduced mitochondrial respiration, autophagy, abnormal RBP assembly	E102Q	NA
*ERBB4*	Growth factor, receptor tyrosine kinase	Reduced neuregulin phosphorylation and activation	R927Q, R1275W	cancer
*ANXA11*	calcium-dependent phospholipid-binding protein	abnormal protein aggregation and loss of intracellular calcium responses, abnormal stress granules	D40G, G228Lfs*29, H390P or R456H	FTD
*CREST*	calcium-dependent activator of transcription	Possible inflammatory mechanisms	Q388X	cancer
*DAO*	Brain D-serine regulation, dopamine synthesis	D-serine accumulation in the spinal cord reduced neuroprotection	R199W	Bipolar diseases

**Table 3:** Examples of proteomic markers with potential application in ALS diagnosis and prognosis.

Marker	Subjects	Biofluids	Results	Use of Marker	Reference
NFL	44 ALS, 20 FTD, 20AD, 19 PD, 6 CJD	serum	NFL levels were elevated in ALS patients and positively correlated with disease progression and shorter survival. Therefore, a cut-off level was established for ALS diagnosis.	Differential diagnosis, prognosis	[188]
NFL	45 sporadic ALS, 21 OCNSDs, 19 MPN, 14 NIMPN and 19 controls	CSF	CSF-NFL may not be useful in differential diagnosis, but a positive correlation was found between disease progression. Negative correlation with ALS function scores.	Differential diagnosis, disease progression	[184]
NFL	171 ALS, 60 mimic-ALS	Plasma, CSF	Both plasma and CSF NFL levels were higher in ALS patients than in mimic-ALS patients. In addition, rapidly progressive ALS and short survival were associated with higher plasma and CSF NFL levels.	Differential diagnosis, prognosis	[189]
NFH and NFL	234 ALS, 44 mimic-ALS	Plasma, CSF	CSF NFH and plasma NFL were higher in ALS patients compared to non-ALS. Plasma NFL may be useful to distinguish the long-and-short survival patients.	Differential diagnosis, prognosis	[190]
pNFH	135 c9orf72 carriers (28 controls, 86 ALS 21 FTD) 107 non-carrier (37 controls, 45ALS, 25 FTD)	CSF	In c9orf72 carriers, pNFH levels were higher, especially in patients with rapid progression. Higher pNFH levels were also associated with shorter survival in C9orf72 carrier ALS patients.	Disease course prediction, prognosis	[205]
pNFH	85 ALS patients, 31 mimic-ALS, 215 controls	CSF, serum	Serum and CSF pNFH were correlated with disease duration, and symptoms of pNFH in serum did not correlate with motor neuron dysfunctions.	Disease progression	[191]
NFH	136 ALS 104 controls	plasma	Higher NFH levels were associated with rapid progression and shorter duration in the early stage but not in the late stage.	Disease progression, prognosis	[192]
TDP43	30 ALS 29 controls	CSF	TDP increases in patients’ CSF, especially in the early disease stage.	Disease onset prediction	[208]
TDP43	36 ALS, 30 PD, 24 controls	CSF	CSF-TDP43 may be useful in the differential diagnosis from ALS.	Differential diagnosis	[209]
TDP43	C9orf72 carriers and non-carriers, healthy and non-ALS controls	CSF	Loss of TDP43 splicing in c9orf72 carriers, even before clinical symptoms.	Disease onset prediction	[214]
Tau	57 ALS, 110 non-ALS	CSF	No significant difference between ALS and controls in CSF-Tau.	NA	[200]
t-Tau, p-Tau, NFL	85 ALS, 30 ALS mimic 51 other NDD	CSF	The p-Tau/t-Tau ratio was lower in ALS patients compared to ALS mimic and other NDDs. High t-Tau and NFL possibly associated with lower survival.	Differential diagnosis, prognosis	[201]
t-Tau, TDP43 NFL	75 ALS patients	plasma	NFL and Tau predictive markers for rapid ALS progression.	Disease progression	[202]
p-Tau/T-Tau	NA	Meta-analysis	Increased t-Tau and reduced pTau/tTau ratio in ALS may be promising candidates.	Disease diagnosis, prognosis	[203]
poly-GP (c9orf72)	*C9orf72* carriers (27 asymptomatic, 83 ALS/FTD, 24 other), non-carriers (48 controls, 57 ALS, 15 others)	CSF, blood	Elevated poly(GP) proteins were detected in the CSF of ALS and asymptomatic c9orf72 repeat expansion carriers. CSF levels of poly(GP) remained constant during the disease course, and they reflected the responses to ASO treatments.	Disease onset prediction, therapy response	[215]
poly(GA), poly(GR)	Healthy individuals and *C9orf72* carriers	CSF	CSF poly(GA) and poly(GR) levels were reduced during ASO treatment.	Disease onset prediction, therapy response	[216]
SOD1	*SOD1* carrier ALS patients	CSF	Reduced levels of mutant SOD1 in CSF after ASO treatment. Reduced mutant SOD1 expression (mRNA).	Disease onset prediction, therapy response	[217]
SOD1	93 ALS, 88 controls, 89 other NDD	CSF	Reduced mutant SOD1 expression (mRNA). CSF SOD levels were higher in ALS patients. Potentially a good pharmacodynamic marker.	Disease onset prediction, therapy response	[218]
cytokines	812 ALS, 639 controls	Meta-analysis	Elevated levels of TNF alpha, TNFR1, IL6, IL1beta, meta-analysis in ALS patients.	Diagnosis	[195]
cytokines	95 ALS, 88 controls	Blood	Several pro-inflammatory markers (such as IL-2, IL-8, and TNF alpha) were elevated. IFN gamma reductions.	Diagnosis	[196]
cytokines	79 ALS 79 controls	Plasma	Many cytokines are elevated in ALS. IL6 is potentially the most effective marker for ALS diagnosis.	Diagnosis	[197]
cytokines	10 ALS 10 controls	Serum	Reduced antioxidant, malondialdehyde and 8-hydroxy-2′-deoxyguanosine level in serum. Elevated GSSG/GSH ratio, IL6, and IL8 levels.	Diagnosis	[198]
106 cytokines, GFs, BB markers	ALS patients in controls	Serum	Several markers (such as fractalkine, BDNF, EGF, PDGF, Dkk-1, MIF, angiopoietin-2 or S100β) may be segregated with ALS. Serum proteins reflect peripheral rather than CNS biofluids.	diagnosis	[199]
CHIT1, YKL-40 MCP-1	105 ALS, 102 disease control, 16 ALS-mimic	CSF	CHIT1 and YKL-40 correlated with disease progression. CHIT1 levels were higher in patients with motor neuron degeneration.	Disease progression, prognosis	[210]
chitinases	82 ALS, 10 ALS-mimic, 10 PLS, 25 controls	CSF	Chitinases were elevated with ALS compared with control and correlated with disease progression.	Disease progression, diagnosis	[219]
CHIT1	316 patients (ALS, ALS mimic, FTD, AD, PD and controls)	CSF	CHIT1 was elevated in ALS patients. Useful marker in microglia and macrophage activation.	Disease progression	[220]
MMPs and TIMPs	30ALS 15 controls	CSF, serum	MT-MMP-1, MMP-2, MMP-9, and TIMP-1 expression was increased in ALS, and MMP9 decreased. In addition, a correlation was found between MMPs, TIMPs, and disease duration.	Disease progression, prognosis	[221]
MMPS	30 ALS, 15 controls	Serum	MT-MMP-1 and MMP-9 potential application in differentiating ALS and controls.	Disease diagnosis, prognosis	[213]
SPP1 and COL6A1	763 ALS, 703 controls	plasma	Elevated fibroblast markers were associated with shorter survival.	Disease prognosis	[222]

## Data Availability

Not applicable.

## Abbreviations

8-OHdG	8-hydroxy-2′-deoxyguanosine
AAV	adeno-associated virus
AD	Alzheimer’s disease
ALS	Amyotrophic lateral sclerosis
ALS2	alsin
ANG	angiogenin
ANXA11	Annexin 11
ASO	antisense oligonucleotides
ATP6v1g1	ATPase H+ Transporting V1 Subunit G1
ATXN2	ataxin2
BD	bipolar disease
BDNF	Brain-derived neurotrophic factor
C1QC	subcomponent subunit C
C9orf72	chromosome 9 open reading frame 72
CAP-Gly	N-terminal glycine-rich and cytoskeleton-associated protein
Ccl2	C-C Motif Chemokine Ligand 2
CCDC190	coiled-coil domain-containing protein 19, mitochondrial
CCNF	Cyclin F
CHCHD2	Coiled-coil-helix-coiled-coil-helix domain containing 2
CHCHD10	Coiled-coil-helix-coiled-coil-helix domain containing 10
CHIT1	Chitotriosidase-1
CHI3L1 and 2	chitinase-3-like-1 and 2
CHMP2B	Charged multivesicular body protein 2
CK1	casein kinase 1
CMT	Charcot-Marie-Tooth
CNVs	copy number variants
COL6A1	Collagen Type VI Alpha 1 Chain
COX	Cyclooxygenase
CREST	Calcium-Responsive Transactivator
CRP	C-Reactive Protein
CRYBB2	Crystallin Beta B2
CRYBB3	Crystallin Beta B3
CRYBB2P1	Crystallin Beta B2 Pseudogene 1
CSF	cerebrospinal fluid
Cxcl10	C-X-C motif chemokine ligand 10
DAO	D-Amino Acid Oxidase
DCTN1	Dynactin 1
DPP6	dipeptidyl-peptidase 6
DPR	dipeptide repeat protein
EGF	Epidermal Growth Factor
ESCRT-III	Endosomal Sorting Complex Required For Transport III
ER	endoplasmic reticulum
ERBB4 or HER4	Erb-B2 Receptor Tyrosine Kinase 4
EV	extracellular vehicles
fALS	familial ALS
FIG4	phosphoinositide 5-phosphatase
FTD	frontotemporal dementia
FUS	Fused In Sarcoma
Fs	frameshift
GOF	gain-of-function toxicity
GSH	glutathione
GSSG	glutathione disulfide
HD	Huntington’s disease
hnRNP	heterogeneous nuclear ribonucleoparticle
HNRNPA	Heterogeneous Nuclear Ribonucleoprotein A1
HREM	hexanucleotide repeat expansion mutation
HSP	hereditary spastic paraplegia
HSPB1	chaperon small heat-shock protein family B member 1
IBM	Inclusion-Body Myositis
IL	interleukin
IP3	the inositol trisphosphate receptor
LC3	Microtubule-associated protein 1A/1B-light chain 3
LRP5L	LDL Receptor Related Protein 5 Like
LMNs	lower motor neurons
LOF	loss-of-function
KEAP1	Kelch-like ECH-associated protein 1
MATR3	Matrin3
MCP-1	monocyte chemoattractant protein-1
MDA	malondialdehyde
MMN	multifocal motor neuropathy
MMP	matrix metalloproteases
MND	motor neuron diseases
MSP	multisystem proteinopathy
MT-MMP	membrane type-matrix metalloproteinase
MVBs	multivesicular bodies
NES	nuclear export signal motif
NEK1	NMA-related kinase 1
NEFH	Neurofilament Heavy Chain
NEFM	Neurofilament Modest Chain
NFs	neurofilaments
NFHs	neurofilament heavy chain
NFLs	neurofilament light chain
NFMs	neurofilament medium chain
NFE2L2/Nrf2	nuclear erythroid derived 2, Nuclear Factor Erythroid 2-Related Factor 2
NIPA	NIPA Magnesium Transporter 1
NIR	INHAT repressor
NLS	nuclear localization signal
NMDA	N-methyl-D-aspartate
NOC2L	nucleolar complex protein 2 homolog
NRGs	neuregulin proteins
OPA1	optic atrophy 1
OPTN	optineurin
PD	Parkinson’s disease
PDGF	Platelet-derived growth factor
PDCD6IP	programmed cell death 6-interacting protein
PFN1	Profilin-1
PMA	progressive muscular atrophy
polyQ	Polyglutamine
PRPH	Peripherin
PRKN	parkin
PTPRZ1	receptor-type tyrosine-protein phosphatase zeta
RRM	RNA recognition motifs
RGG box	arginine-glycine rich box
ROS	reactive oxygen species
sALS	sporadic ALS
SBMA	spinal and bulbar muscular atrophy
SERPINA3	alpha-1-antichymotrypsin
SETX	Sentaxin
SIGMAR1	Sigma Non-Opioid Intracellular Receptor 1
SMNs	survival motor neurons
SOD1	Cu–Zn superoxide dismutase
SPG11	Spastacin
SPP1	secreted phosphoprotein 1
SQSTM	Sequestosome 1
SSR	subsynaptic reticulum
TAF15	TATA-box-binding protein-associated factor 15
TARDBP or TDP43	TAR DNA Binding Protein 43
TAS	total antioxidant status
TBK1	TANK Binding Kinase 1
TIMP	tissue inhibitor of metalloproteinases
TNF	tumor necrosis factor
TUBA4A	Tubulin Alpha 4a
UBQLN2	Ubiquilin-2
UMN	upper motor neurons
UPF1	RNA Helicase and ATPase
UPF3b	Up-Frameshift Suppressor 3 Homolog B
UPS	ubiquitin-proteasome system
VAPB	vesicle-associated membrane-protein-associated protein B
VCAN	versican core protein
VCP	Valosin Containing Protein
YKL-40	chitinase-3-like protein 1

## References

[1] Masrori P., Van Damme P. (2020). Amyotrophic lateral sclerosis: A clinical review. Eur. J. Neurol..

[2] Hulisz D. (2018). Amyotrophic lateral sclerosis: Disease state overview. Am. J. Manag. Care.

[3] Ravits J.M., La Spada A.R. (2009). ALS motor phenotype heterogeneity, focality, and spread: Deconstructing motor neuron degeneration. Neurology.

[4] Testa D., Lovati R., Ferrarini M., Salmoiraghi F., Filippini G. (2004). Survival of 793 patients with amyotrophic lateral sclerosis diagnosed over a 28-year period. Amyotroph. Lateral Scler. Other Mot. Neuron Disord..

[5] Logroscino G., Traynor B.J., Hardiman O., Chiò A., Mitchell D., Swingler R.J., Millul A., Benn E., Beghi E., Eurals F. (2010). Incidence of amyotrophic lateral sclerosis in Europe. J. Neurol. Neurosurg. Psychiatry.

[6] Chio A., Logroscino G., Hardiman O., Swingler R., Mitchell D., Beghi E., Traynor B. (2009). Eurals Consortium Prognostic factors in ALS: A critical review. Amyotroph. Lateral Scler..

[7] Walhout R., Verstraete E., Heuvel M.P.V.D., Veldink J.H., Berg L.H.V.D. (2018). Patterns of symptom development in patients with motor neuron disease. Amyotroph. Lateral Scler. Front. Degener..

[8] Burrell J.R., Kiernan M.C., Vucic S., Hodges J.R. (2011). Motor neuron dysfunction in frontotemporal dementia. Brain.

[9] Abramzon Y.A., Fratta P., Traynor B.J., Chia R. (2020). The Overlapping Genetics of Amyotrophic Lateral Sclerosis and Frontotemporal Dementia. Front. Neurosci..

[10] Ghasemi M., Brown R.H. (2018). Genetics of Amyotrophic Lateral Sclerosis. Cold Spring Harb. Perspect. Med..

[11] Karch C.M., Wen N., Fan C.C., Yokoyama J.S., Kouri N., Ross O., Höglinger G., Müller U., Ferrari R., Hardy J. (2018). Selective Genetic Overlap Between Amyotrophic Lateral Sclerosis and Diseases of the Frontotemporal Dementia Spectrum. JAMA Neurol..

[12] Fang F., Ingre C., Roos P.M., Kamel F., Piehl F. (2015). Risk factors for amyotrophic lateral sclerosis. Clin. Epidemiology.

[13] Chernyshova K., Inoue K., Yamashita S.-I., Fukuchi T., Kanki T. (2019). Glaucoma-Associated Mutations in the Optineurin Gene Have Limited Impact on Parkin-Dependent Mitophagy. Investig. Opthalmology Vis. Sci..

[14] Nekoua M.P., Mercier A., Alhazmi A., Sane F., Alidjinou E.K., Hober D. (2022). Fighting Enteroviral Infections to Prevent Type 1 Diabetes. Microorganisms.

[15] Singh A.K., Kapoor V., Thotala D., Hallahan D.E. (2020). TAF15 contributes to the radiation-inducible stress response in cancer. Oncotarget.

[16] Yao L., Cen J., Pan J., Liu D., Wang Y., Chen Z., Ruan C., Chen S. (2017). TAF15–ZNF384 fusion gene in childhood mixed phenotype acute leukemia. Cancer Genet..

[17] Silverman J.M., Fernando S.M., Grad L.I., Hill A.F., Turner B.J., Yerbury J.J., Cashman N.R. (2016). Disease Mechanisms in ALS: Misfolded SOD1 Transferred through Exosome-Dependent and Exosome-Independent Pathways. Cell. Mol. Neurobiol..

[18] Mejzini R., Flynn L.L., Pitout I.L., Fletcher S., Wilton S.D., Akkari P.A. (2019). ALS Genetics, Mechanisms, and Therapeutics: Where Are We Now?. Front Neurosci..

[19] Kaur S.J., McKeown S.R., Rashid S. (2016). Mutant SOD1 mediated pathogenesis of Amyotrophic Lateral Sclerosis. Gene.

[20] Huai J., Zhang Z. (2019). Structural Properties and Interaction Partners of Familial ALS-Associated SOD1 Mutants. Front. Neurol..

[21] Wallace M.A., Liou L.L., Martins J., Clement M.H., Bailey S., Longo V.D., Valentine J.S., Gralla E.B. (2004). Superoxide inhibits 4Fe-4S cluster enzymes involved in amino acid biosynthesis. Cross-compartment protection by CuZn-superoxide dismutase. J. Biol. Chem..

[22] De Souza P.V.S., Pinto W.B.V.R., Farias I.B., Badia B.M.L., Pinto I.F.N., Costa G.C., Marin C.M., Dos Santos Jorge A.C., Souto E.C., Serrano P.L. (2021). Progressive spastic tetraplegia and axial hypotonia (STAHP) due to SOD1 deficiency: Is it really a new entity?. Orphanet J. Rare Dis..

[23] Orrell R.W. (2000). Amyotrophic lateral sclerosis: Copper/zinc superoxide dismutase (SOD1) gene mutations. Neuromuscul. Disord..

[24] Wang L.-Q., Ma Y., Yuan H.-Y., Zhao K., Zhang M.-Y., Wang Q., Huang X., Xu W.-C., Dai B., Chen J. (2022). Cryo-EM structure of an amyloid fibril formed by full-length human SOD1 reveals its conformational conversion. Nat. Commun..

[25] Broom H.R., Rumfeldt J.A.O., Vassall K.A., Meiering E.M. (2015). Destabilization of the dimer interface is a common consequence of diverse ALS-associated mutations in metal free SOD1. Protein Sci..

[26] Kabashi E., Valdmanis P.N., Dion P., Rouleau G.A. (2007). Oxidized/misfolded superoxide dismutase-1: The cause of all amyotrophic lateral sclerosis?. Ann. Neurol..

[27] Peggion C., Scalcon V., Massimino M.L., Nies K., Lopreiato R., Rigobello M.P., Bertoli A. (2022). SOD1 in ALS: Taking Stock in Pathogenic Mechanisms and the Role of Glial and Muscle Cells. Antioxidants.

[28] Saeed M., Yang Y., Deng H.-X., Hung W.-Y., Siddique N., Dellefave L., Gellera C., Andersen P.M., Siddique T. (2009). Age and founder effect of SOD1 A4V mutation causing ALS. Neurology.

[29] Valentine J.S., Hart P.J. (2003). Misfolded CuZnSOD and amyotrophic lateral sclerosis. Proc. Natl. Acad. Sci. USA.

[30] Zou Z., Liu M.-S., Li X.-G., Cui L.-Y. (2016). H46R SOD1 mutation is consistently associated with a relatively benign form of amyotrophic lateral sclerosis with slow progression. Amyotroph. Lateral Scler. Front. Degener..

[31] Holmøy T., Bjørgo K., Roos P.M. (2007). Slowly Progressing Amyotrophic Lateral Sclerosis Caused by H46R SOD1 Mutation. Eur. Neurol..

[32] Antonyuk S., Elam J.S., Hough M.A., Strange R.W., Doucette P.A., Rodriguez J.A., Hayward L.J., Valentine J.S., Hart P.J., Hasnain S.S. (2005). Structural consequences of the familial amyotrophic lateral sclerosis SOD1 mutant His46Arg. Protein Sci..

[33] Kreilaus F., Guerra S., Masanetz R., Menne V., Yerbury J., Karl T. (2020). Novel behavioural characteristics of the superoxide dismutase 1 G93A (SOD1G93A) mouse model of amyotrophic lateral sclerosis include sex-dependent phenotypes. Genes Brain Behav..

[34] Hensley K., Mhatre M., Mou S., Pye Q.N., Stewart C., West M., Williamson K.S. (2006). On the relation of oxidative stress to neuroinflammation: Lessons learned from the G93A-SOD1 mouse model of amyotrophic lateral sclerosis. Antioxid. Redox Signal..

[35] Sephton C.F., Cenik B., Cenik B.K., Herz J., Yu G. (2012). TDP-43 in central nervous system development and function: Clues to TDP-43-associated neurodegeneration. Biol. Chem..

[36] Jo M., Lee S., Jeon Y.-M., Kim S., Kwon Y., Kim H.-J. (2020). The role of TDP-43 propagation in neurodegenerative diseases: Integrating insights from clinical and experimental studies. Exp. Mol. Med..

[37] Peng C., Trojanowski J.Q., Lee V.M.-Y. (2020). Protein transmission in neurodegenerative disease. Nat. Rev. Neurol..

[38] Buratti E., Baralle F.E. (2012). TDP-43: Gumming up neurons through protein-protein and protein-RNA interactions. Trends Biochem. Sci..

[39] Wang P., Deng J., Dong J., Liu J., Bigio E.H., Mesulam M., Wang T., Sun L., Wang L., Lee A.Y.-L. (2019). TDP-43 induces mitochondrial damage and activates the mitochondrial unfolded protein response. PLoS Genet..

[40] Jiang L., Ngo S.T. (2022). Altered TDP-43 Structure and Function: Key Insights into Aberrant RNA, Mitochondrial, and Cellular and Systemic Metabolism in Amyotrophic Lateral Sclerosis. Metabolites.

[41] Liu W., Li C., Shan J., Wang Y., Chen G. (2021). Insights into the aggregation mechanism of RNA recognition motif domains in TDP-43: A theoretical exploration. R. Soc. Open Sci..

[42] Lim L., Wei Y., Lu Y., Song J. (2016). ALS-Causing Mutations Significantly Perturb the Self-Assembly and Interaction with Nucleic Acid of the Intrinsically Disordered Prion-Like Domain of TDP-43. PLoS Biol..

[43] Tan R.H., Ke Y.D., Ittner L.M., Halliday G.M. (2017). ALS/FTLD: Experimental models and reality. Acta Neuropathol..

[44] Johnson B.S., Snead D., Lee J.J., McCaffery J.M., Shorter J., Gitler A.D. (2009). TDP-43 is intrinsically aggregation-prone, and amyotrophic lateral sclerosis-linked mutations accelerate aggregation and increase toxicity. J. Biol. Chem..

[45] Mitsuzawa S., Akiyama T., Nishiyama A., Suzuki N., Kato M., Warita H., Izumi R., Osana S., Koyama S., Kato T. (2018). TARDBP p.G376D mutation, found in rapid progressive familial ALS, induces mislocalization of TDP-43. Eneurologicalsci.

[46] Lattante S., Rouleau G.A., Kabashi E. (2013). TARDBP and FUS mutations associated with amyotrophic lateral sclerosis: Summary and update. Hum. Mutat..

[47] Pesiridis G.S., Lee V.M.-Y., Trojanowski J.Q. (2009). Mutations in TDP-43 link glycine-rich domain functions to amyotrophic lateral sclerosis. Hum. Mol. Genet..

[48] Chen C., Ding X., Akram N., Xue S., Luo S.-Z. (2019). Fused in Sarcoma: Properties, Self-Assembly and Correlation with Neurodegenerative Diseases. Molecules.

[49] Ho W.Y., Agrawal I., Tyan S.-H., Sanford E., Chang W.-T., Lim K., Ong J., Tan B.S.Y., Moe A.A.K., Yu R. (2021). Dysfunction in nonsense-mediated decay, protein homeostasis, mitochondrial function, and brain connectivity in ALS-FUS mice with cognitive deficits. Acta Neuropathol. Commun..

[50] Deng H., Gao K., Jankovic J. (2014). The role of FUS gene variants in neurodegenerative diseases. Nat. Rev. Neurol..

[51] An H., Skelt L., Notaro A., Highley J.R., Fox A.H., La Bella V., Buchman V.L., Shelkovnikova T.A. (2019). ALS-linked FUS mutations confer loss and gain of function in the nucleus by promoting excessive formation of dysfunctional paraspeckles. Acta Neuropathol. Commun..

[52] Shang Y., Huang E.J. (2016). Mechanisms of FUS mutations in familial amyotrophic lateral sclerosis. Brain Res..

[53] Scotter E.L., Chen H.-J., Shaw C.E. (2015). TDP-43 Proteinopathy and ALS: Insights into Disease Mechanisms and Therapeutic Targets. Neurotherapeutics.

[54] Sama R.R.K., Fallini C., Gatto R., McKeon J.E., Song Y., Rotunno M.S., Penaranda S., Abdurakhmanov I., Landers J.E., Morfini G. (2017). ALS-linked FUS exerts a gain of toxic function involving aberrant p38 MAPK activation. Sci. Rep..

[55] Zhou Y., Liu S., Liu G., Öztürk A., Hicks G.G. (2013). ALS-associated FUS mutations result in compromised FUS alternative splicing and autoregulation. PLoS Genet..

[56] Kamelgarn M., Chen J., Kuang L., Jin H., Kasarskis E.J., Zhu H. (2018). ALS mutations of FUS suppress protein translation and disrupt the regulation of nonsense-mediated decay. Proc. Natl. Acad. Sci. USA.

[57] Neumann M., Rademakers R., Roeber S., Baker M., Kretzschmar H.A., Mackenzie I.R.A. (2009). A new subtype of frontotemporal lobar degeneration with FUS pathology. Brain.

[58] King O.D., Gitler A.D., Shorter J. (2012). The tip of the iceberg: RNA-binding proteins with prion-like domains in neurodegenerative disease. Brain Res..

[59] Nakaya T., Maragkakis M. (2018). Amyotrophic Lateral Sclerosis associated FUS mutation shortens mitochondria and induces neurotoxicity. Sci. Rep..

[60] Yu M., Zhao X., Wu W., Wang Q., Liu J., Zhang W., Yuan Y., Hong D., Wang Z., Deng J. (2022). Widespread Mislocalization of FUS Is Associated with Mitochondrial Abnormalities in Skeletal Muscle in Amyotrophic Lateral Sclerosis With FUS Mutations. J. Neuropathol. Exp. Neurol..

[61] Smeyers J., Banchi E.G., Latouche M. (2021). C9ORF72: What It Is, What It Does, and Why It Matters. Front Cell Neurosci..

[62] DeJesus-Hernandez M., Mackenzie I.R., Boeve B.F., Boxer A.L., Baker M., Rutherford N.J., Nicholson A.M., Finch N.A., Flynn H., Adamson J. (2011). Expanded GGGGCC hexanucleotide repeat in noncoding region of C9ORF72 causes chromosome 9p-linked FTD and ALS. Neuron.

[63] Renton A.E., Majounie E., Waite A., Simón-Sánchez J., Rollinson S., Gibbs J.R., Schymick J.C., Laaksovirta H., van Swieten J.C., Myllykangas L. (2011). A hexanucleotide repeat expansion in C9ORF72 is the cause of chromosome 9p21-linked ALS-FTD. Neuron.

[64] Gijselinck I., Van Mossevelde S., van der Zee J., Sieben A., Engelborghs S., De Bleecker J., Ivanoiu A., Deryck O., Edbauer D., Zhang M. (2015). The C9orf72 repeat size correlates with onset age of disease, DNA methylation and transcriptional downregulation of the promoter. Mol. Psychiatry.

[65] Van der Zee J., Gijselinck I., Dillen L., Van Langenhove T., Theuns J., Engelborghs S., Philtjens S., Vandenbulcke M., Sleegers K., Sieben A. (2013). European Early-Onset Dementia Consortium. A pan-European study of the C9orf72 repeat associated with FTLD: Geographic prevalence, genomic instability, and intermediate repeats. Hum. Mutat..

[66] Koppers M., Blokhuis A.M., Westeneng H., Terpstra M.L., Zundel C.A.C., de Sá R.V., Schellevis R.D., Waite A.J., Blake D.J., Veldink J.H. (2015). C 9orf72 ablation in mice does not cause motor neuron degeneration or motor deficits. Ann. Neurol..

[67] Mori K., Weng S.-M., Arzberger T., May S., Rentzsch K., Kremmer E., Schmid B., Kretzschmar H.A., Cruts M., Van Broeckhoven C. (2013). The C9orf72 GGGGCC repeat is translated into aggregating dipeptide-repeat proteins in FTLD/ALS. Science.

[68] Quaegebeur A., Glaria I., Lashley T., Isaacs A.M. (2020). Soluble and insoluble dipeptide repeat protein measurements in C9orf72-frontotemporal dementia brains show regional differential solubility and correlation of poly-GR with clinical severity. Acta Neuropathol. Commun..

[69] May S., Hornburg D., Schludi M.H., Arzberger T., Rentzsch K., Schwenk B.M., Grässer F.A., Mori K., Kremmer E., Banzhaf-Strathmann J. (2014). C9orf72 FTLD/ALS-associated Gly-Ala dipeptide repeat proteins cause neuronal toxicity and Unc119 sequestration. Acta Neuropathol..

[70] Mizielinska S., Grönke S., Niccoli T., Ridler C.E., Clayton E.L., Devoy A., Moens T., Norona F.E., Woollacott I.O.C., Pietrzyk J. (2014). C9orf72 repeat expansions cause neurodegeneration in Drosophila through arginine-rich proteins. Science.

[71] Haeusler A.R., Donnelly C.J., Rothstein J.D. (2016). The expanding biology of the C9orf72 nucleotide repeat expansion in neurodegenerative disease. Nat. Rev. Neurosci..

[72] Chitiprolu M., Jagow C., Tremblay V., Bondy-Chorney E., Paris G., Savard A., Palidwor G., Barry F.A., Zinman L., Keith J. (2018). A complex of C9ORF72 and p62 uses arginine methylation to eliminate stress granules by autophagy. Nat. Commun..

[73] Walker C., Herranz-Martin S., Karyka E., Liao C., Lewis K., Elsayed W., Lukashchuk V., Chiang S.-C., Ray S., Mulcahy P.J. (2017). C9orf72 expansion disrupts ATM-mediated chromosomal break repair. Nat. Neurosci..

[74] Mead R.J., Shan N., Reiser H.J., Marshall F., Shaw P.J. (2023). Amyotrophic lateral sclerosis: A neurodegenerative disorder poised for successful therapeutic translation. Nat. Rev. Drug Discov..

[75] Ugbode C., West R.J. (2021). Lessons learned from CHMP2B, implications for frontotemporal dementia and amyotrophic lateral sclerosis. Neurobiol. Dis..

[76] Babst M., Katzmann D.J., Estepa-Sabal E.J., Meerloo T., Emr S.D. (2002). Escrt-III: An endosome-associated heterooligomeric protein complex required for mvb sorting. Dev. Cell.

[77] Deng X., Sun X., Yue W., Duan Y., Hu R., Zhang K., Ni J., Cui J., Wang Q., Chen Y. (2022). CHMP2B regulates TDP-43 phosphorylation and cytotoxicity independent of autophagy via CK1. J. Cell Biol..

[78] Koppers M., van Blitterswijk M.M., Vlam L., Rowicka P.A., van Vught P.W., Groen E.J., Spliet W.G., Engelen-Lee J., Schelhaas H.J., de Visser M. (2012). VCP mutations in familial and sporadic amyotrophic lateral sclerosis. Neurobiol. Aging.

[79] Ferrari V., Cristofani R., Tedesco B., Crippa V., Chierichetti M., Casarotto E., Cozzi M., Mina F., Piccolella M., Galbiati M. (2022). Valosin Containing Protein (VCP): A Multistep Regulator of Autophagy. Int. J. Mol. Sci..

[80] Shaw C.E. (2010). Capturing VCP: Another Molecular Piece in the ALS Jigsaw Puzzle. Neuron.

[81] Johnson J.O., Mandrioli J., Benatar M., Abramzon Y., Van Deerlin V.M., Trojanowski J.Q., Gibbs J.R., Brunetti M., Gronka S., Wuu J. (2010). Exome sequencing reveals VCP mutations as a cause of familial ALS. Neuron.

[82] Cadoni M.P.L., Biggio M.L., Arru G., Secchi G., Orrù N., Clemente M.G., Sechi G., Yamoah A., Tripathi P., Orrù S. (2020). VAPB ER-Aggregates, A Possible New Biomarker in ALS Pathology. Cells.

[83] Landers J.E., Leclerc A.L., Shi L., Virkud A., Cho T., Maxwell M.M., Henry A.F., Polak M., Glass J.D., Kwiatkowski T.J. (2008). New VAPB deletion variant and exclusion of VAPB mutations in familial ALS. Neurology.

[84] Nicholson G., Lenk G.M., Reddel S.W., Grant A.E., Towne C.F., Ferguson C.J., Simpson E., Scheuerle A., Yasick M., Hoffman S. (2011). Distinctive genetic and clinical features of CMT4J: A severe neuropathy caused by mutations in the PI(3,5)P_2_ phosphatase FIG4. Brain.

[85] Kon T., Mori F., Tanji K., Miki Y., Toyoshima Y., Yoshida M., Sasaki H., Kakita A., Takahashi H., Wakabayashi K. (2014). ALS-associated protein FIG4 is localized in Pick and Lewy bodies, and also neuronal nuclear inclusions, in polyglutamine and intranuclear inclusion body diseases. Neuropathology.

[86] Osmanovic A., Rangnau I., Kosfeld A., Abdulla S., Janssen C., Auber B., Raab P., Preller M., Petri S., Weber R.G. (2017). FIG4 variants in central European patients with amyotrophic lateral sclerosis: A whole-exome and targeted sequencing study. Eur. J. Hum. Genet..

[87] Bertolin C., Querin G., Bozzoni V., Martinelli I., De Bortoli M., Rampazzo A., Gellera C., Pegoraro E., Sorarù G. (2018). New FIG4 gene mutations causing aggressive ALS. Eur. J. Neurol..

[88] Liu C.-Y., Lin J.-L., Feng S.-Y., Che C.-H., Huang H.-P., Zou Z.-Y. (2022). Novel Variants in the *FIG4* Gene Associated with Chinese Sporadic Amyotrophic Lateral Sclerosis with Slow Progression. J. Clin. Neurol..

[89] Khani M., Shamshiri H., Fatehi F., Rohani M., Ashtiani B.H., Akhoundi F.H., Alavi A., Moazzeni H., Taheri H., Ghani M.T. (2020). Description of combined ARHSP/JALS phenotype in some patients with SPG11 mutations. Mol. Genet. Genom. Med..

[90] Boutry M., Pierga A., Matusiak R., Branchu J., Houllegatte M., Ibrahim Y., Balse E., El Hachimi K.-H., Brice A., Stevanin G. (2019). Loss of spatacsin impairs cholesterol trafficking and calcium homeostasis. Commun. Biol..

[91] Blauw H.M., van Rheenen W., Koppers M., Van Damme P., Waibel S., Lemmens R., van Vught P.W.J., Meyer T., Schulte C., Gasser T. (2012). NIPA1 polyalanine repeat expansions are associated with amyotrophic lateral sclerosis. Hum. Mol. Genet..

[92] Goytain A., Hines R.M., El-Husseini A., Quamme G.A. (2007). NIPA1(SPG6), the basis for autosomal dominant form of hereditary spastic paraplegia, encodes a functional Mg2+ transporter. J. Biol. Chem..

[93] Corrado L., Brunetti M., Di Pierro A., Barberis M., Croce R., Bersano E., De Marchi F., Zuccalà M., Barizzone N., Calvo A. (2019). Analysis of the GCG repeat length in NIPA1 gene in C9orf72-mediated ALS in a large Italian ALS cohort. Neurol Sci..

[94] Tazelaar G.H., Dekker A.M., van Vugt J.J., van der Spek R.A., Westeneng H.-J., Kool L.J., Kenna K.P., van Rheenen W., Pulit S.L., McLaughlin R.L. (2019). Association of NIPA1 repeat expansions with amyotrophic lateral sclerosis in a large international cohort. Neurobiol. Aging.

[95] Williams K.L., Topp S., Yang S., Smith B., Fifita J.A., Warraich S.T., Zhang K.Y., Farrawell N., Vance C., Hu X. (2016). CCNF mutations in amyotrophic lateral sclerosis and frontotemporal dementia. Nat. Commun..

[96] Rayner S.L., Hogan A., Davidson J.M., Cheng F., Luu L., Morsch M., Blair I., Chung R., Lee A. (2022). Cyclin F, Neurodegeneration, and the Pathogenesis of ALS/FTD. Neuroscientist.

[97] Farrawell N.E., Bax M., McAlary L., McKenna J., Maksour S., Do-Ha D., Rayner S.L., Blair I.P., Chung R.S., Yerbury J.J. (2023). ALS-linked CCNF variant disrupts motor neuron ubiquitin homeostasis. Hum. Mol. Genet..

[98] Yu Y., Nakagawa T., Morohoshi A., Nakagawa M., Ishida N., Suzuki N., Aoki M., Nakayama K. (2019). Pathogenic mutations in the ALS gene CCNF cause cytoplasmic mislocalization of Cyclin F and elevated VCP ATPase activity. Hum. Mol. Genet..

[99] Deng H.-X., Chen W., Hong S.-T., Boycott K.M., Gorrie G.H., Siddique N., Yang Y., Fecto F., Shi Y., Zhai H. (2011). Mutations in UBQLN2 cause dominant X-linked juvenile and adult-onset ALS and ALS/dementia. Nature.

[100] Chang L., Monteiro M.J. (2015). Defective Proteasome Delivery of Polyubiquitinated Proteins by Ubiquilin-2 Proteins Containing ALS Mutations. PLoS ONE.

[101] Wu J.J., Cai A., Greenslade J.E., Higgins N.R., Fan C., Le N.T., Tatman M., Whiteley A.M., Prado M.A., Dieriks B.V. (2021). ALS/FTD mutations in UBQLN2 impede autophagy by reducing autophagosome acidification through loss of function. Proc. Natl. Acad. Sci. USA.

[102] Capponi S., Geuens T., Geroldi A., Origone P., Verdiani S., Cichero E., Adriaenssens E., De Winter V., di Poggio M.B., Barberis M. (2016). Molecular Chaperones in the Pathogenesis of Amyotrophic Lateral Sclerosis: The Role of HSPB1. Hum. Mutat..

[103] Heilman P.L., Song S., Miranda C.J., Meyer K., Srivastava A.K., Knapp A., Wier C.G., Kaspar B.K., Kolb S.J. (2017). HSPB1 mutations causing hereditary neuropathy in humans disrupt non-cell autonomous protection of motor neurons. Exp. Neurol..

[104] Haidar M., Asselbergh B., Adriaenssens E., De Winter V., Timmermans J.P., Auer-Grumbach M., Juneja M., Timmerman V. (2019). Neuropathy-causing mutations in HSPB1 impair autophagy by disturbing the formation of SQSTM1/p62 bodies. Autophagy.

[105] Dierick I., Irobi J., Janssens S., Theuns J., Lemmens R., Jacobs A., Corsmit E., Hersmus N., Van Den Bosch L., Robberecht W. (2007). Genetic variant in the HSPB1 promoter region impairs the HSP27 stress response. Hum Mutat. 2007 Aug;28(8):830. Hum Mutat..

[106] Komatsu M., Waguri S., Koike M., Sou Y.S., Ueno T., Hara T., Mizushima N., Iwata J., Ezaki J., Murata S. (2007). Homeostatic levels of p62 control cytoplasmic inclusion body formation in autophagy-deficient mice. Cell.

[107] Goode A., Butler K., Long J., Cavey J., Scott D., Shaw B., Sollenberger J., Gell C., Johansen T., Oldham N.J. (2016). Defective recognition of LC3B by mutant SQSTM1/p62 implicates impairment of autophagy as a pathogenic mechanism in ALS-FTLD. Autophagy.

[108] Deng Z., Lim J., Wang Q., Purtell K., Wu S., Palomo G.M., Tan H., Manfredi G., Zhao Y., Peng J. (2020). ALS-FTLD-linked mutations of SQSTM1/p62 disrupt selective autophagy and NFE2L2/NRF2 anti-oxidative stress pathway. Autophagy.

[109] Xiao Y., Zou Q., Xie X., Liu T., Li H.S., Jie Z., Jin J., Hu H., Manyam G., Zhang L. (2017). The kinase TBK1 functions in dendritic cells to regulate T cell homeostasis, autoimmunity, and antitumor immunity. J. Exp. Med..

[110] Harding O., Evans C.S., Ye J., Cheung J., Maniatis T., Holzbaur E.L.F. (2021). ALS- and FTD-associated missense mutations in TBK1 differentially disrupt mitophagy. Proc. Natl. Acad. Sci. USA.

[111] Ye J., Cheung J., Gerbino V., Ahlsén G., Zimanyi C., Hirsh D., Maniatis T. (2019). Effects of ALS-associated TANK binding kinase 1 mutations on protein–protein interactions and kinase activity. Proc. Natl. Acad. Sci. USA.

[112] Toth R.P., Atkin J.D. (2018). Dysfunction of Optineurin in Amyotrophic Lateral Sclerosis and Glaucoma. Front. Immunol..

[113] Wild P., Farhan H., McEwan D.G., Wagner S., Rogov V.V., Brady N.R., Richter B., Korac J., Waidmann O., Choudhary C. (2011). Phosphorylation of the autophagy receptor optineurin restricts Salmonella growth. Science.

[114] Richter B., Sliter D.A., Herhaus L., Stolz A., Wang C., Beli P., Zaffagnini G., Wild P., Martens S., Wagner S.A. (2016). Phosphorylation of OPTN by TBK1 enhances its binding to Ub chains and promotes selective autophagy of damaged mitochondria. Proc. Natl. Acad. Sci. USA.

[115] Li Y., Kang J., Horwitz M.S. (1998). Interaction of an adenovirus E3 14.7-kilodalton protein with a novel tumor necrosis factor alpha-inducible cellular protein containing leucine zipper domains. Mol. Cell. Biol..

[116] Maruyama H., Morino H., Ito H., Izumi Y., Kato H., Watanabe Y., Kinoshita Y., Kamada M., Nodera H., Suzuki H. (2010). Mutations of optineurin in amyotrophic lateral sclerosis. Nature.

[117] Ayloo S., Lazarus J.E., Dodda A., Tokito M., Ostap E.M., Holzbaur E.L. (2014). Dynactin functions as both a dynamic tether and brake during dynein-driven motility. Nat. Commun..

[118] Vilarino-Guell C., Wider C., Soto-Ortolaza A.I., Cobb S.A., Kachergus J.M., Keeling B.H., Dachsel J.C., Hulihan M.M., Dickson D.W., Wszolek Z.K. (2009). Characterization of DCTN1 genetic variability in neurodegeneration. Neurology.

[119] Deshimaru M., Kinoshita-Kawada M., Kubota K., Watanabe T., Tanaka Y., Hirano S., Ishidate F., Hiramoto M., Ishikawa M., Uehara Y. (2021). DCTN1 Binds to TDP-43 and Regulates TDP-43 Aggregation. Int. J. Mol. Sci..

[120] Thiel C., Kessler K., Giessl A., Dimmler A., Shalev S.A., von der Haar S., Zenker M., Zahnleiter D., Stöss H., Beinder E. (2011). NEK1 mutations cause short-rib polydactyly syndrome type majewski. Am. J. Hum. Genet..

[121] Kenna K.P., van Doormaal P.T., Dekker A.M., Ticozzi N., Kenna B.J., Diekstra F.P., van Rheenen W., van Eijk K.R., Jones A.R., Keagle P. (2016). NEK1 variants confer susceptibility to amyotrophic lateral sclerosis. Nat. Genet..

[122] Brenner D., Müller K., Wieland T., Weydt P., Böhm S., Lulé D., Hübers A., Neuwirth C., Weber M., Borck G. (2016). *NEK1* mutations in familial amyotrophic lateral sclerosis. Brain.

[123] Wu C.-H., Fallini C., Ticozzi N., Keagle P.J., Sapp P.C., Piotrowska K., Lowe P., Koppers M., McKenna-Yasek D., Baron D.M. (2012). Mutations in the profilin 1 gene cause familial amyotrophic lateral sclerosis. Nature.

[124] Yang C., Danielson E.W., Qiao T., Metterville J., Brown R.H., Landers J.E., Xu Z. (2016). Mutant PFN1 causes ALS phenotypes and progressive motor neuron degeneration in mice by a gain of toxicity. Proc. Natl. Acad. Sci. USA.

[125] Schmidt E.J., Funes S., McKeon J.E., Morgan B.R., Boopathy S., O’connor L.C., Bilsel O., Massi F., Jégou A., Bosco D.A. (2021). ALS-linked PFN1 variants exhibit loss and gain of functions in the context of formin-induced actin polymerization. Proc. Natl. Acad. Sci. USA.

[126] Teyssou E., Chartier L., Roussel D., Perera N.D., Nemazanyy I., Langui D., Albert M., Larmonier T., Saker S., Salachas F. (2022). The Amyotrophic Lateral Sclerosis M114T PFN1 Mutation Deregulates Alternative Autophagy Pathways and Mitochondrial Homeostasis. Int. J. Mol. Sci..

[127] Romano R., Del Fiore V.S., Bucci C. (2022). Role of the Intermediate Filament Protein Peripherin in Health and Disease. Int. J. Mol. Sci..

[128] Castellanos-Montiel M.J., Chaineau M., Durcan T.M. (2020). The Neglected Genes of ALS: Cytoskeletal Dynamics Impact Synaptic Degeneration in ALS. Front. Cell. Neurosci..

[129] Corrado L., Carlomagno Y., Falasco L., Mellone S., Godi M., Cova E., Cereda C., Testa L., Mazzini L., D’Alfonso S. (2011). A novel peripherin gene (PRPH) mutation identified in one sporadic amyotrophic lateral sclerosis patient. Neurobiol. Aging.

[130] Robertson J., Doroudchi M.M., Nguyen M.D., Durham H.D., Strong M.J., Shaw G., Julien J.-P., Mushynski W.E. (2003). A neurotoxic peripherin splice variant in a mouse model of ALS. J. Cell Biol..

[131] Campos-Melo D., Hawley Z.C.E., Strong M.J. (2018). Dysregulation of human NEFM and NEFH mRNA stability by ALS-linked miRNAs. Mol. Brain.

[132] Mol M.O., Wong T.H., Melhem S., Basu S., Viscusi R., Galjart N., Rozemuller A.J., Fallini C., Landers J.E., Kaat L.D. (2021). Novel TUBA4A Variant Associated with Familial Frontotemporal Dementia. Neurol. Genet..

[133] Smith B.N., Ticozzi N., Fallini C., Gkazi A.S., Topp S., Kenna K.P., Scotter E.L., Kost J., Keagle P., Miller J.W. (2014). Exome-wide rare variant analysis identifies TUBA4A mutations associated with familial ALS. Neuron.

[134] Rademakers R., van Blitterswijk M. (2014). Excess of rare damaging TUBA4A variants suggests cytoskeletal defects in ALS. Neuron.

[135] Daneshmandpour Y., Bahmanpour Z., Kazeminasab S., Moghadam E.A., Alehabib E., Chapi M., Tafakhori A., Aghaei N., Darvish H., Emamalizadeh B. (2022). A novel mutation in the ALS2 gene in an iranian kurdish family with juvenile amyotrophic lateral sclerosis. Amyotroph. Lateral Scler. Front. Degener..

[136] Hand C.K., Devon R.S., Gros-Louis F., Rochefort D., Khoris J., Meininger V., Bouchard J.-P., Camu W., Hayden M.R., Rouleau G.A. (2003). Mutation Screening of the ALS2 Gene in Sporadic and Familial Amyotrophic Lateral Sclerosis. Arch. Neurol..

[137] Kim J., Kim S., Nahm M., Li T.-N., Lin H.-C., Kim Y.D., Lee J., Yao C.-K., Lee S. (2021). ALS2 regulates endosomal trafficking, postsynaptic development, and neuronal survival. J. Cell Biol..

[138] Chen Y.-Z., Hashemi S.H., Anderson S.K., Huang Y., Moreira M.-C., Lynch D.R., Glass I.A., Chance P.F., Bennett C.L. (2006). Senataxin, the yeast Sen1p orthologue: Characterization of a unique protein in which recessive mutations cause ataxia and dominant mutations cause motor neuron disease. Neurobiol. Dis..

[139] Ma L., Shi Y., Chen Z., Li S., Zhang J. (2018). A novel SETX gene mutation associated with Juvenile amyotrophic lateral sclerosis. Brain Behav..

[140] Kapeli K., Pratt G.A., Vu A.Q., Hutt K.R., Martinez F.J., Sundararaman B., Batra R., Freese P., Lambert N.J., Huelga S.C. (2016). Distinct and shared functions of ALS-associated proteins TDP-43, FUS and TAF15 revealed by multisystem analyses. Nat. Commun..

[141] Ticozzi N., Vance C., LeClerc A., Keagle P., Glass J., McKenna-Yasek D., Sapp P., Silani V., Bosco D., Shaw C. (2011). Mutational analysis reveals the FUS homolog TAF15 as a candidate gene for familial amyotrophic lateral sclerosis. Am. J. Med Genet. Part B Neuropsychiatr. Genet..

[142] Aluri K.C., Salisbury J.P., Prehn J.H.M., Agar J.N. (2020). Loss of angiogenin function is related to earlier ALS onset and a paradoxical increase in ALS duration. Sci. Rep..

[143] Bradshaw W.J., Rehman S., Pham T.T.K., Thiyagarajan N., Lee R.L., Subramanian V., Acharya K.R. (2017). Structural insights into human angiogenin variants implicated in Parkinson’s disease and Amyotrophic Lateral Sclerosis. Sci. Rep..

[144] Sproviero W., Shatunov A., Stahl D., Shoai M., van Rheenen W., Jones A.R., Al-Sarraj S., Andersen P.M., Bonini N.M., Conforti F.L. (2017). ATXN2 trinucleotide repeat length correlates with risk of ALS. Neurobiol. Aging.

[145] Elden A.C., Kim H.J., Hart M.P., Chen-Plotkin A.S., Johnson B.S., Fang X., Armakola M., Geser F., Greene R., Lu M.M. (2010). Ataxin-2 intermediate-length polyglutamine expansions are associated with increased risk for ALS. Nature.

[146] Beijer D., Kim H.J., Guo L., O’donovan K., Mademan I., Deconinck T., Van Schil K., Fare C.M., Drake L.E., Ford A.F. (2021). Characterization of HNRNPA1 mutations defines diversity in pathogenic mechanisms and clinical presentation. J. Clin. Investig..

[147] Honda H., Hamasaki H., Wakamiya T., Koyama S., Suzuki S.O., Fujii N., Iwaki T. (2014). Loss of hnRNPA1 in ALS spinal cord motor neurons with TDP-43-positive inclusions. Neuropathology.

[148] Deshaies J.-E., Shkreta L., Moszczynski A.J., Sidibé H., Semmler S., Fouillen A., Bennett E.R., Bekenstein U., Destroismaisons L., Toutant J. (2018). TDP-43 regulates the alternative splicing of hnRNP A1 to yield an aggregation-prone variant in amyotrophic lateral sclerosis. Brain.

[149] Fratta P., Isaacs A.M. (2018). The snowball effect of RNA binding protein dysfunction in amyotrophic lateral sclerosis. Brain.

[150] Malik A.M., Barmada S.J. (2021). Matrin 3 in neuromuscular disease: Physiology and pathophysiology. J. Clin. Investig..

[151] Johnson J.O., Pioro E.P., Boehringer A., Chia R., Feit H., Renton A.E., Pliner H.A., Abramzon Y., Marangi G., Winborn B.J. (2014). Mutations in the Matrin 3 gene cause familial amyotrophic lateral sclerosis. Nat. Neurosci..

[152] Boehringer A., Garcia-Mansfield K., Singh G., Bakkar N., Pirrotte P., Bowser R. (2017). ALS Associated Mutations in Matrin 3 Alter Protein-Protein Interactions and Impede mRNA Nuclear Export. Sci. Rep..

[153] Jankovic M., Novakovic I., Dawod P.G.A., Dawod A.G.A., Drinic A., Motaleb F.I.A., Ducic S., Nikolic D. (2021). Current Concepts on Genetic Aspects of Mitochondrial Dysfunction in Amyotrophic Lateral Sclerosis. Int. J. Mol. Sci..

[154] Bannwarth S., Ait-El-Mkadem S., Chaussenot A., Genin E.C., Lacas-Gervais S., Fragaki K., Berg-Alonso L., Kageyama Y., Serre V., Moore D.G. (2014). A mitochondrial origin for frontotemporal dementia and amyotrophic lateral sclerosis through CHCHD10 involvement. Brain.

[155] Ruan Y., Hu J., Che Y., Liu Y., Luo Z., Cheng J., Han Q., He H., Zhou Q. (2022). CHCHD2 and CHCHD10 regulate mitochondrial dynamics and integrated stress response. Cell Death Dis..

[156] Straub I.R., Weraarpachai W., A Shoubridge E. (2021). Multi-OMICS study of a CHCHD10 variant causing ALS demonstrates metabolic rewiring and activation of endoplasmic reticulum and mitochondrial unfolded protein responses. Hum. Mol. Genet..

[157] Genin E.C., Hounoum B.M., Bannwarth S., Fragaki K., Lacas-Gervais S., Mauri-Crouzet A., Lespinasse F., Neveu J., Ropert B., Augé G. (2019). Mitochondrial defect in muscle precedes neuromuscular junction degeneration and motor neuron death in CHCHD10S59L/+ mouse. Acta Neuropathol..

[158] Genin E.C., Bannwarth S., Lespinasse F., Ortega-Vila B., Fragaki K., Itoh K., Villa E., Lacas-Gervais S., Jokela M., Auranen M. (2018). Loss of MICOS complex integrity and mitochondrial damage, but not TDP-43 mitochondrial localisation, are likely associated with severity of CHCHD10-related diseases. Neurobiol. Dis..

[159] Straub I.R., Janer A., Weraarpachai W., Zinman L., Robertson J., Rogaeva E., Shoubridge E.A. (2018). Loss of CHCHD10-CHCHD2 complexes required for respiration underlies the pathogenicity of a CHCHD10 mutation in ALS. Hum. Mol. Genet..

[160] Brockmann S.J., Freischmidt A., Oeckl P., Müller K., Ponna S.K., Helferich A.M., Paone C., Reinders J., Kojer K., Orth M. (2018). CHCHD10 mutations p.R15L and p.G66V cause motoneuron disease by haploinsufficiency. Hum. Mol. Genet..

[161] Hayashi T., Su T.-P. (2007). Sigma-1 Receptor Chaperones at the ER- Mitochondrion Interface Regulate Ca^2+^ Signaling and Cell Survival. Cell.

[162] Fukunaga K., Shinoda Y., Tagashira H. (2015). The role of SIGMAR1 gene mutation and mitochondrial dysfunction in amyotrophic lateral sclerosis. J. Pharmacol. Sci..

[163] Dreser A., Vollrath J.T., Sechi A., Johann S., Roos A., Yamoah A., Katona I., Bohlega S., Wiemuth D., Tian Y. (2017). The ALS-linked E102Q mutation in Sigma receptor-1 leads to ER stress-mediated defects in protein homeostasis and dysregulation of RNA-binding proteins. Cell Death Differ..

[164] Takahashi Y., Fukuda Y., Yoshimura J., Toyoda A., Kurppa K., Moritoyo H., Belzil V.V., Dion P.A., Higasa K., Doi K. (2013). ERBB4 mutations that disrupt the neuregulin-ErbB4 pathway cause amyotrophic lateral sclerosis type 19. Am. J. Hum. Genet..

[165] Wang F., Liu X., He J., Zhang N., Chen L., Tang L., Fan D. (2022). Analysis of ERBB4 Variants in Amyotrophic Lateral Sclerosis Within a Chinese Cohort. Front. Neurol..

[166] Nahm M., Lim S.M., Kim Y.-E., Park J., Noh M.-Y., Lee S., Roh J.E., Hwang S.-M., Park C.-K., Kim Y.H. (2020). *ANXA11* mutations in ALS cause dysregulation of calcium homeostasis and stress granule dynamics. Sci. Transl. Med..

[167] Wang Y., Duan X., Zhou X., Wang R., Zhang X., Cao Z., Wang X., Zhou Z., Sun Y., Peng D. (2022). ANXA11 mutations are associated with amyotrophic lateral sclerosis-frontotemporal dementia. Front. Neurol..

[168] Sung W., Nahm M., Lim S.M., Noh M.-Y., Lee S., Hwang S.-M., Kim Y.H., Park J., Oh K.-W., Ki C.-S. (2022). Clinical and genetic characteristics of amyotrophic lateral sclerosis patients with *ANXA11* variants. Brain Commun..

[169] Cheng C., Yang K., Wu X., Zhang Y., Shan S., Gitler A., Ghosh A., Qiu Z. (2019). Loss of CREST leads to neuroinflammatory responses and ALS-like motor defects in mice. Transl. Neurodegener..

[170] Kondori N.R., Paul P., Robbins J.P., Liu K., Hildyard J.C.W., Wells D.J., De Belleroche J.S. (2018). Focus on the Role of D-serine and D-amino Acid Oxidase in Amyotrophic Lateral Sclerosis/Motor Neuron Disease (ALS). Front. Mol. Biosci..

[171] Mitchell J., Paul P., Chen H.-J., Morris A., Payling M., Falchi M., Habgood J., Panoutsou S., Winkler S., Tisato V. (2010). Familial amyotrophic lateral sclerosis is associated with a mutation in D-amino acid oxidase. Proc. Natl. Acad. Sci. USA.

[172] Cronin S., Blauw H.M., Veldink J.H., van Es M.A., Ophoff R.A., Bradley D.G., Berg L.H.v.D., Hardiman O. (2008). Analysis of genome-wide copy number variation in Irish and Dutch ALS populations. Hum. Mol. Genet..

[173] Wang X.-B., Cui N.-H., Gao J.-J., Qiu X.-P., Zheng F. (2014). SMN1 duplications contribute to sporadic amyotrophic lateral sclerosis susceptibility: Evidence from a meta-analysis. J. Neurol. Sci..

[174] Corcia P., Camu W., Halimi J.M., Vourc’h P., Antar C., Vedrine S., Giraudeau B., de Toffol B., Andres C.R., French ALS Study Group (2006). SMN1 gene, but not SMN2, is a risk factor for sporadic ALS. Neurology.

[175] Moisse M., Zwamborn R.A.J., Vugt J., Spek R., Rheenen W., Kenna B., Van Eijk K., Kenna K., Corcia P., Couratier P. (2021). The Effect of *SMN* Gene Dosage on ALS Risk and Disease Severity. Ann. Neurol..

[176] Blauw H.M., Veldink J.H., van Es M.A., van Vught P.W., Saris C.G., van der Zwaag B., Franke L., Burbach J.P., Wokke J.H., Ophoff R.A. (2008). Copy-number variation in sporadic amyotrophic lateral sclerosis: A genome-wide screen. Lancet Neurol..

[177] Blauw H.M., Al-Chalabi A., Andersen P.M., van Vught P.W., Diekstra F.P., van Es M.A., Saris C.G., Groen E.J., van Rheenen W., Koppers M. (2010). A large genome scan for rare CNVs in amyotrophic lateral sclerosis. Hum. Mol. Genet..

[178] Robelin L., De Aguilar J.L.G. (2014). Blood Biomarkers for Amyotrophic Lateral Sclerosis: Myth or Reality?. BioMed Res. Int..

[179] Tarasiuk J., Kułakowska A., Drozdowski W., Kornhuber J., Lewczuk P. (2012). CSF markers in amyotrophic lateral sclerosis. J. Neural Transm..

[180] Raghunathan R., Turajane K., Wong L.C. (2022). Biomarkers in Neurodegenerative Diseases: Proteomics Spotlight on ALS and Parkinson’s Disease. Int. J. Mol. Sci..

[181] Porter L., Shoushtarizadeh A., Jelinek G.A., Brown C.R., Lim C.K., De Livera A.M., Jacobs K.R., Weiland T.J. (2020). Metabolomic Biomarkers of Multiple Sclerosis: A Systematic Review. Front. Mol. Biosci..

[182] Calvo A.C., Pradat P.-F., Mendonça D.M.F., Manzano R. (2014). Decoding amyotrophic lateral sclerosis: Discovery of novel disease-related biomarkers and future perspectives in neurodegeneration. BioMed Res. Int..

[183] Pradat P.-F., Dubourg O., de Tapia M., di Scala F., Dupuis L., Lenglet T., Bruneteau G., Salachas F., Lacomblez L., Corvol J.-C. (2011). Muscle gene expression is a marker of amyotrophic lateral sclerosis severity. Neurodegener. Dis..

[184] Sun Q., Zhao X., Li S., Yang F., Wang H., Cui F., Huang X. (2020). CSF Neurofilament Light Chain Elevation Predicts ALS Severity and Progression. Front. Neurol..

[185] Tortelli R., Copetti M., Ruggieri M., Cortese R., Capozzo R., Leo A., D’Errico E., Mastrapasqua M., Zoccolella S., Pellegrini F. (2015). Cerebrospinal fluid neurofilament light chain levels: Marker of progression to generalized amyotrophic lateral sclerosis. Eur. J. Neurol..

[186] King A.E., Blizzard C.A., Southam K.A., Vickers J.C., Dickson T.C. (2012). Degeneration of axons in spinal white matter in G93A mSOD1 mouse characterized by NFL and α-internexin immunoreactivity. Brain Res..

[187] Forgrave L.M., Ma M., Best J.R., DeMarco M.L. (2019). The diagnostic performance of neurofilament light chain in CSF and blood for Alzheimer’s disease, frontotemporal dementia, and amyotrophic lateral sclerosis: A systematic review and meta-analysis. Alzheimer’s Dement..

[188] Verde F., Steinacker P., Weishaupt J.H., Kassubek J., Oeckl P., Halbgebauer S., Tumani H., von Arnim C.A.F., Dorst J., Feneberg E. (2019). Neurofilament light chain in serum for the diagnosis of amyotrophic lateral sclerosis. J. Neurol. Neurosurg. Psychiatry.

[189] Vacchiano V., Mastrangelo A., Zenesini C., Masullo M., Quadalti C., Avoni P., Polischi B., Cherici A., Capellari S., Salvi F. (2021). Plasma and CSF Neurofilament Light Chain in Amyotrophic Lateral Sclerosis: A Cross-Sectional and Longitudinal Study. Front. Aging Neurosci..

[190] Behzadi A., Pujol-Calderón F., Tjust A.E., Wuolikainen A., Höglund K., Forsberg K., Portelius E., Blennow K., Zetterberg H., Andersen P.M. (2021). Neurofilaments can differentiate ALS subgroups and ALS from common diagnostic mimics. Sci. Rep..

[191] De Schaepdryver M., Jeromin A., Gille B., Claeys K., Herbst V., Brix B., Van Damme P., Poesen K. (2018). Comparison of elevated phosphorylated neurofilament heavy chains in serum and cerebrospinal fluid of patients with amyotrophic lateral sclerosis. J. Neurol. Neurosurg. Psychiatry.

[192] Lu C.-H., Petzold A., Topping J., Allen K., Macdonald-Wallis C., Clarke J., Pearce N., Kuhle J., Giovannoni G., Fratta P. (2015). Plasma neurofilament heavy chain levels and disease progression in amyotrophic lateral sclerosis: Insights from a longitudinal study. J. Neurol. Neurosurg. Psychiatry.

[193] De Marchi F., Munitic I., Amedei A., Berry J.D., Feldman E.L., Aronica E., Nardo G., Van Weehaeghe D., Niccolai E., Prtenjaca N. (2021). Interplay between immunity and amyotrophic lateral sclerosis: Clinical impact. Neurosci. Biobehav. Rev..

[194] Hu Y., Cao C., Qin X.-Y., Yu Y., Yuan J., Zhao Y., Cheng Y. (2017). Increased peripheral blood inflammatory cytokine levels in amyotrophic lateral sclerosis: A meta-analysis study. Sci. Rep..

[195] Lu C.-H., Allen K., Oei F., Leoni E., Kuhle J., Tree T., Fratta P., Sharma N., Sidle K., Howard R. (2016). Systemic inflammatory response and neuromuscular involvement in amyotrophic lateral sclerosis. Neurol. Neuroimmunol. Neuroinflamm..

[196] Michaelson N., Facciponte D., Bradley W., Stommel E. (2017). Cytokine expression levels in ALS: A potential link between inflammation and BMAA-triggered protein misfolding. Cytokine Growth Factor Rev..

[197] Tortelli R., Zecca C., Piccininni M., Benmahamed S., Dell’Abate M.T., Barulli M.R., Capozzo R., Battista P., Logroscino G. (2020). Plasma Inflammatory Cytokines Are Elevated in ALS. Front. Neurol..

[198] Blasco H., Garcon G., Patin F., Veyrat-Durebex C., Boyer J., Devos D., Vourc’h P., Andres C.R., Corcia P. (2017). Panel of Oxidative Stress and Inflammatory Biomarkers in ALS: A Pilot Study. Can. J. Neurol. Sci./J. Can. des Sci. Neurol..

[199] Cao M.C., Cawston E.E., Chen G., Brooks C., Douwes J., McLean D., Graham E.S., Dragunow M., Scotter E.L. (2022). Serum biomarkers of neuroinflammation and blood-brain barrier leakage in amyotrophic lateral sclerosis. BMC Neurol..

[200] Paladino P., Valentino F., Piccoli T., Piccoli F., La Bella V. (2009). Cerebrospinal fluid tau protein is not a biological marker in amyotrophic lateral sclerosis. Eur. J. Neurol..

[201] Scarafino A., D’errico E., Introna A., Fraddosio A., Distaso E., Tempesta I., Morea A., Mastronardi A., Leante R., Ruggieri M. (2018). Diagnostic and prognostic power of CSF Tau in amyotrophic lateral sclerosis. J. Neurol..

[202] Kojima Y., Kasai T., Noto Y.-I., Ohmichi T., Tatebe H., Kitaoji T., Tsuji Y., Kitani-Morii F., Shinomoto M., Allsop D. (2021). Amyotrophic lateral sclerosis: Correlations between fluid biomarkers of NfL, TDP-43, and tau, and clinical characteristics. PLoS ONE.

[203] Thapa S., Bhattarai A., Shah S., Chand S., Bagherieh S., Mirmosayyeb O., Mishra S.K. (2023). Diagnostic Role of Tau Proteins in Amyotrophic Lateral Sclerosis: A Systematic Review and Meta-Analysis. Acta Neurologica. Scandinavica..

[204] Sun J., Carrero J.J., Zagai U., Evans M., Ingre C., Pawitan Y., Fang F. (2020). Blood biomarkers and prognosis of amyotrophic lateral sclerosis. Eur. J. Neurol..

[205] Gendron T.F., Daughrity L.M., Heckman M.G., Diehl N.N., Wuu J., Miller T.M., Pastor P., Trojanowski J.Q., Grossman M., Berry J.D. (2017). Phosphorylated neurofilament heavy chain: A biomarker of survival for *C9ORF 72* -associated amyotrophic lateral sclerosis. Ann. Neurol..

[206] Gaiani A., Martinelli I., Bello L., Querin G., Puthenparampil M., Ruggero S., Toffanin E., Cagnin A., Briani C., Pegoraro E. (2017). Diagnostic and Prognostic Biomarkers in Amyotrophic Lateral Sclerosis: Neurofilament Light Chain Levels in Definite Subtypes of Disease. JAMA Neurol..

[207] Jamerlan A.M., Shim K.H., Youn Y.C., Teunissen C., An S.S.A., Scheltens P., Kim S. (2023). Increased oligomeric TDP-43 in the plasma of Korean frontotemporal dementia patients with semantic dementia. Alzheimer’s Dement..

[208] Kasai T., Tokuda T., Ishigami N., Sasayama H., Foulds P., Mitchell D.J., Mann D.M.A., Allsop D., Nakagawa M. (2009). Increased TDP-43 protein in cerebrospinal fluid of patients with amyotrophic lateral sclerosis. Acta Neuropathol..

[209] Beyer L., Günther R., Koch J.C., Klebe S., Hagenacker T., Lingor P., Biesalski A.S., Hermann A., Nabers A., Gold R. (2021). TDP-43 as structure-based biomarker in amyotrophic lateral sclerosis. Ann. Clin. Transl. Neurol..

[210] Gille B., De Schaepdryver M., Dedeene L., Goossens J., Claeys K.G., Van Den Bosch L., Tournoy J., Van Damme P., Poesen K. (2019). Inflammatory markers in cerebrospinal fluid: Independent prognostic biomarkers in amyotrophic lateral sclerosis?. J. Neurol. Neurosurg. Psychiatry.

[211] Demestre M., Parkin-Smith G., Petzold A., Pullen A. (2005). The pro and the active form of matrix metalloproteinase-9 is increased in serum of patients with amyotrophic lateral sclerosis. J. Neuroimmunol..

[212] Łukaszewicz-Zając M., Mroczko B., Słowik A. (2014). Matrix metalloproteinases (MMPs) and their tissue inhibitors (TIMPs) in amyotrophic lateral sclerosis (ALS). J. Neural Transm..

[213] Sokołowska B., Jóźwik A., Niebroj-Dobosz I., Janik P., Kwiecinski H. (2009). Evaluation of matrix metalloproteinases in serum of patients with amyotrophic lateral sclerosis with pattern recognition methods. J. Physiol. Pharmacol..

[214] Irwin K.E., Jasin P., Braunstein K.E., Sinha I., Bowden K.D., Moghekar A., Oh E.S., Raitcheva D., Bartlett D., Berry J.D. (2023). A fluid biomarker reveals loss of TDP-43 splicing repression in pre-symptomatic ALS. bioRxiv.

[215] Gendron T.F., Chew J., Stankowski J.N., Hayes L.R., Zhang Y.-J., Prudencio M., Carlomagno Y., Daughrity L.M., Jansen-West K., Perkerson E.A. (2017). Poly(GP) proteins are a useful pharmacodynamic marker for *C9ORF72* -associated amyotrophic lateral sclerosis. Sci. Transl. Med..

[216] Krishnan G., Raitcheva D., Bartlett D., Prudencio M., McKenna-Yasek D.M., Douthwright C., Oskarsson B.E., Ladha S., King O.D., Barmada S.J. (2022). Poly(GR) and poly(GA) in cerebrospinal fluid as potential biomarkers for C9ORF72-ALS/FTD. Nat. Commun..

[217] Gertsman I., Wuu J., McAlonis-Downes M., Ghassemian M., Ling K., Rigo F., Bennett F., Benatar M., Miller T.M., Da Cruz S. (2019). An endogenous peptide marker differentiates SOD1 stability and facilitates pharmacodynamic monitoring in SOD1 amyotrophic lateral sclerosis. JCI Investig..

[218] Winer L., Srinivasan D., Chun S., Lacomis D., Jaffa M., Fagan A., Holtzman D.M., Wancewicz E., Bennett C.F., Bowser R. (2013). SOD1 in Cerebral Spinal Fluid as a Pharmacodynamic Marker for Antisense Oligonucleotide Therapy. JAMA Neurol..

[219] Thompson A.G., Gray E., Bampton A., Raciborska D., Talbot K., Turner M.R. (2019). CSF chitinase proteins in amyotrophic lateral sclerosis. J. Neurol. Neurosurg. Psychiatry.

[220] Steinacker P., Verde F., Fang L., Feneberg E., Oeckl P., Roeber S., Anderl-Straub S., Danek A., Diehl-Schmid J., Fassbender K. (2018). Chitotriosidase (CHIT1) is increased in microglia and macrophages in spinal cord of amyotrophic lateral sclerosis and cerebrospinal fluid levels correlate with disease severity and progression. J. Neurol. Neurosurg. Psychiatry.

[221] Niebroj-Dobosz I., Janik P., Sokołowska B., Kwiecinski H. (2010). Matrix metalloproteinases and their tissue inhibitors in serum and cerebrospinal fluid of patients with amyotrophic lateral sclerosis. Eur. J. Neurol..

[222] Månberg A., Skene N., Sanders F., Trusohamn M., Remnestål J., Szczepińska A., Aksoylu I.S., Lönnerberg P., Ebarasi L., Wouters S. (2021). Altered perivascular fibroblast activity precedes ALS disease onset. Nat. Med..

[223] Gaur N., Perner C., Witte O.W., Grosskreutz J. (2020). The Chitinases as Biomarkers for Amyotrophic Lateral Sclerosis: Signals From the CNS and Beyond. Front. Neurol..

[224] Mizielinska S., Lashley T., Norona F.E., Clayton E.L., Ridler C.E., Fratta P., Isaacs A.M. (2013). C9orf72 frontotemporal lobar degeneration is characterised by frequent neuronal sense and antisense RNA foci. Acta Neuropathol..

[225] Benatar M., Wuu J., Andersen P.M., Lombardi V., Malaspina A. (2018). Neurofilament light: A candidate biomarker of presymptomatic amyotrophic lateral sclerosis and phenoconversion. Ann. Neurol..

[226] Benigni M., Ricci C., Jones A.R., Giannini F., Al-Chalabi A., Battistini S. (2016). Identification of miRNAs as Potential Biomarkers in Cerebrospinal Fluid from Amyotrophic Lateral Sclerosis Patients. NeuroMolecular Med..

[227] Barbo M., Ravnik-Glavač M. (2023). Extracellular Vesicles as Potential Biomarkers in Amyotrophic Lateral Sclerosis. Genes.

[228] Ding X., Ma M., Teng J., Teng R.K., Zhou S., Yin J., Fonkem E., Huang J.H., Wu E., Wang X. (2015). Exposure to ALS-FTD-CSF generates TDP-43 aggregates in glioblastoma cells through exosomes and TNTs-like structure. Oncotarget.

[229] An Y.R., Hwang S.Y. (2014). Toxicology study with microRNA. Mol. Cell. Toxicol..

[230] Kim H., Pyo J.Y., Moon J., Lee S., Kim M., Choi Y., Shin D.-I., Park B.-G. (2022). Identification of miRNA expression associated with Alzheimer’s disease and neurodegeneration in rat models with obstructive sleep apnea. Mol. Cell. Toxicol..

[231] Campos-Melo D., Droppelmann C.A., He Z., Volkening K., Strong M.J. (2013). Altered microRNA expression profile in amyotrophic lateral sclerosis: A role in the regulation of NFL mRNA levels. Mol. Brain.

[232] Chen Y., Wei Q., Chen X., Li C., Cao B., Ou R., Hadano S., Shang H.-F. (2016). Aberration of miRNAs Expression in Leukocytes from Sporadic Amyotrophic Lateral Sclerosis. Front. Mol. Neurosci..

[233] Hayashi N., Doi H., Kurata Y., Kagawa H., Atobe Y., Funakoshi K., Tada M., Katsumoto A., Tanaka K., Kunii M. (2020). Proteomic analysis of exosome-enriched fractions derived from cerebrospinal fluid of amyotrophic lateral sclerosis patients. Neurosci. Res..

[234] De Felice B., Annunziata A., Fiorentino G., Borra M., Biffali E., Coppola C., Cotrufo R., Brettschneider J., Giordana M.L., Dalmay T. (2014). miR-338-3p is over-expressed in blood, CFS, serum and spinal cord from sporadic amyotrophic lateral sclerosis patients. Neurogenetics.

[235] Magen I., Yacovzada N.S., Yanowski E., Coenen-Stass A., Grosskreutz J., Lu C.-H., Greensmith L., Malaspina A., Fratta P., Hornstein E. (2021). Circulating miR-181 is a prognostic biomarker for amyotrophic lateral sclerosis. Nat. Neurosci..

[236] Waller R., Goodall E.F., Milo M., Cooper-Knock J., Da Costa M., Hobson E., Kazoka M., Wollff H., Heath P.R., Shaw P.J. (2017). Serum miRNAs miR-206, 143-3p and 374b-5p as potential biomarkers for amyotrophic lateral sclerosis (ALS). Neurobiol. Aging.

[237] Sproviero D., Gagliardi S., Zucca S., Arigoni M., Giannini M., Garofalo M., Olivero M., Dell’orco M., Pansarasa O., Bernuzzi S. (2021). Different miRNA Profiles in Plasma Derived Small and Large Extracellular Vesicles from Patients with Neurodegenerative Diseases. Int. J. Mol. Sci..

[238] Banack S.A., Dunlop R.A., Stommel E.W., Mehta P., Cox P.A. (2022). miRNA extracted from extracellular vesicles is a robust biomarker of amyotrophic lateral sclerosis. J. Neurol. Sci..

[239] Xiao Y., Wang S.K., Zhang Y., Rostami A., Kenkare A., Casella G., Yuan Z.Q., Li X. (2021). Role of extracellular vesicles in neurodegenerative diseases. Prog. Neurobiol..

[240] McCluskey G., Morrison K.E., Donaghy C., Rene F., Duddy W., Duguez S. (2022). Extracellular Vesicles in Amyotrophic Lateral Sclerosis. Life.

[241] Sproviero D., Gagliardi S., Zucca S., Arigoni M., Giannini M., Garofalo M., Fantini V., Pansarasa O., Avenali M., Ramusino M.C. (2022). Extracellular Vesicles Derived from Plasma of Patients with Neurodegenerative Disease Have Common Transcriptomic Profiling. Front. Aging Neurosci..

[242] Cano A., Ettcheto M., Bernuz M., Puerta R., Esteban de Antonio E., Sánchez-López E., Souto E.B., Camins A., Martí M., Pividori M.I. (2023). Extracellular vesicles, the emerging mirrors of brain physiopathology. Int. J. Biol. Sci..

[243] Chen P.-C., Wu D., Hu C.-J., Chen H.-Y., Hsieh Y.-C., Huang C.-C. (2020). Exosomal TAR DNA-binding protein-43 and neurofilaments in plasma of amyotrophic lateral sclerosis patients: A longitudinal follow-up study. J. Neurol. Sci..

[244] Pasetto L., Callegaro S., Corbelli A., Fiordaliso F., Ferrara D., Brunelli L., Sestito G., Pastorelli R., Bianchi E., Cretich M. (2021). Decoding distinctive features of plasma extracellular vesicles in amyotrophic lateral sclerosis. Mol. Neurodegener..

[245] Pregnolato F., Cova L., Doretti A., Bardelli D., Silani V., Bossolasco P. (2021). Exosome microRNAs in Amyotrophic Lateral Sclerosis: A Pilot Study. Biomolecules.

[246] Chen Y., Xia K., Chen L., Fan D. (2019). Increased Interleukin-6 Levels in the Astrocyte-Derived Exosomes of Sporadic Amyotrophic Lateral Sclerosis Patients. Front. Neurosci..

[247] Dhasmana S., Dhasmana A., Narula A.S., Jaggi M., Yallapu M.M., Chauhan S.C. (2022). The panoramic view of amyotrophic lateral sclerosis: A fatal intricate neurological disorder. Life Sci..

[248] Traynor B.J. (2014). A roadmap for genetic testing in ALS. J. Neurol. Neurosurg. Psychiatry.

[249] Chiò A., Battistini S., Calvo A., Caponnetto C., Conforti F.L., Corbo M., Giannini F., Mandrioli J., Mora G., Sabatelli M. (2014). Genetic counselling in ALS: Facts, uncertainties and clinical suggestions. J. Neurol. Neurosurg. Psychiatry.

[250] Witzel S., Mayer K., Oeckl P. (2022). Biomarkers for amyotrophic lateral sclerosis. Curr. Opin. Neurol..

[251] Rinchetti P., Rizzuti M., Faravelli I., Corti S. (2018). MicroRNA Metabolism and Dysregulation in Amyotrophic Lateral Sclerosis. Mol. Neurobiol..

[252] Liu J., Zhou F., Guan Y., Meng F., Zhao Z., Su Q., Bao W., Wang X., Zhao J., Huo Z. (2022). The Biogenesis of miRNAs and Their Role in the Development of Amyotrophic Lateral Sclerosis. Cells.

[253] Goyal N.A., Berry J.D., Windebank A., Staff N.P., Maragakis N.J., Berg L.H.V.D., Genge A., Miller R., Baloh R.H., Kern R. (2020). Addressing heterogeneity in amyotrophic lateral sclerosis CLINICAL TRIALS. Muscle Nerve.

[254] Beghi E., Mennini T., Bendotti C., Bigini P., Logroscino G., Chio A., Hardiman O., Mitchell D., Swingler R., Traynor B. (2007). The Heterogeneity of Amyotrophic Lateral Sclerosis: A Possible Explanation of Treatment Failure. Curr. Med. Chem..

[255] Leoni E., Bremang M., Mitra V., Zubiri I., Jung S., Lu C.H., Adiutori R., Lombardi V., Russell C., Koncarevic S. (2019). Combined Tissue-Fluid Proteomics to Unravel Phenotypic Variability in Amyotrophic Lateral Sclerosis. Sci. Rep..

[256] Sturmey E., Malaspina A. (2022). Blood biomarkers in ALS: Challenges, applications and novel frontiers. Acta Neurol. Scand..

[257] Kueffner R., Zach N., Bronfeld M., Norel R., Atassi N., Balagurusamy V., Di Camillo B., Chio A., Cudkowicz M., Dillenberger D. (2019). Stratification of amyotrophic lateral sclerosis patients: A crowdsourcing approach. Sci. Rep..

[258] Choi Y.S., Lee M.Y., David A.E., Park Y.S. (2014). Nanoparticles for gene delivery: Therapeutic and toxic effects. Mol. Cell. Toxicol..

[259] Lejman J., Panuciak K., Nowicka E., Mastalerczyk A., Wojciechowska K., Lejman M. (2023). Gene Therapy in ALS and SMA: Advances, Challenges and Perspectives. Int. J. Mol. Sci..

[260] Miller T.M., Cudkowicz M.E., Genge A., Shaw P.J., Sobue G., Bucelli R.C., Chiò A., Van Damme P., Ludolph A.C., Glass J.D. (2022). Trial of Antisense Oligonucleotide Tofersen for *SOD1* ALS. New Engl. J. Med..

[261] Benatar M., Wuu J., Andersen P.M., Bucelli R.C., Andrews J.A., Otto M., Farahany N.A., Harrington E.A., Chen W., Mitchell A.A. (2022). Design of a Randomized, Placebo-Controlled, Phase 3 Trial of Tofersen Initiated in Clinically Presymptomatic SOD1 Variant Carriers: The ATLAS Study. Neurotherapeutics.

[262] Liu Y., Dodart J.-C., Tran H., Berkovitch S., Braun M., Byrne M., Durbin A.F., Hu X.S., Iwamoto N., Jang H.G. (2021). Variant-selective stereopure oligonucleotides protect against pathologies associated with C9orf72-repeat expansion in preclinical models. Nat. Commun..

[263] Smukowski S.N., Maioli H., Latimer C.S., Bird T.D., Jayadev S., Valdmanis P.N. (2022). Progress in Amyotrophic Lateral Sclerosis Gene Discovery: Reflecting on Classic Approaches and Leveraging Emerging Technologies. Neurol. Genet..

[264] Miller T., Cudkowicz M., Shaw P.J., Andersen P.M., Atassi N., Bucelli R.C., Genge A., Glass J., Ladha S., Ludolph A.L. (2020). Phase 1-2 Trial of Antisense Oligonucleotide Tofersen for SOD1 ALS. N. Engl. J. Med..

[265] Arnold C. (2019). Custom therapies pose huge financial burdens. Nat. Med..

[266] Stoica L., Todeasa S.H., Cabrera G.T., Salameh J.S., ElMallah M.K., Mueller C., Brown R.H., Sena-Esteves M. (2016). Adeno-associated virus-delivered artificial microRNA extends survival and delays paralysis in an amyotrophic lateral sclerosis mouse model. Ann. Neurol..

[267] Mueller C., Berry J.D., McKenna-Yasek D.M., Gernoux G., Owegi M.A., Pothier L.M., Douthwright C.L., Gelevski D., Luppino S.D., Blackwood M. (2020). *SOD1* Suppression with Adeno-Associated Virus and MicroRNA in Familial ALS. N. Engl. J. Med..

[268] Fang T., Je G., Pacut P., Keyhanian K., Gao J., Ghasemi M. (2022). Gene Therapy in Amyotrophic Lateral Sclerosis. Cells.

[269] Martier R., Liefhebber J.M., Garcia-Osta A., Miniarikova J., Cuadrado-Tejedor M., Espelosin M., Ursua S., Petry H., van Deventer S.J., Evers M.M. (2019). Targeting RNA-Mediated Toxicity in C9orf72 ALS and/or FTD by RNAi-Based Gene Therapy. Mol. Ther. Nucleic Acids.

[270] Martier R., Liefhebber J.M., Miniarikova J., van der Zon T., Snapper J., Kolder I., Petry H., van Deventer S.J., Evers M.M., Konstantinova P. (2019). Artificial MicroRNAs Targeting C9orf72 Can Reduce Accumulation of Intra-nuclear Transcripts in ALS and FTD Patients. Mol. Ther. Nucleic Acids.

[271] Benatar M., Wuu J. (2012). Presymptomatic studies in ALS: Rationale, challenges, and approach. Neurology.

[272] Benatar M., Turner M.R., Wuu J. (2019). Defining pre-symptomatic amyotrophic lateral sclerosis. Amyotroph. Lateral Scler. Front. Degener..

[273] Corcia P., Lumbroso S., Cazeneuve C., Mouzat K., Camu W., Vourc’h P., on Behalf the FILSLAN network (2020). Pre-symptomatic diagnosis in ALS. Rev. Neurol..

[274] Benatar M., Wuu J., McHutchison C., Postuma R.B., Boeve B.F., Petersen R., A Ross C., Rosen H., Arias J.J., Fradette S. (2022). Preventing amyotrophic lateral sclerosis: Insights from pre-symptomatic neurodegenerative diseases. Brain.

